# *Let-7g* Upregulation Attenuated the KRAS–PI3K–Rac1–Akt Axis-Mediated Bioenergetic Functions

**DOI:** 10.3390/cells12182313

**Published:** 2023-09-19

**Authors:** Kuang-Chen Hung, Ni Tien, Da-Tian Bau, Chun-Hsu Yao, Chan-Hung Chen, Jiun-Long Yang, Meng-Liang Lin, Shih-Shun Chen

**Affiliations:** 1 Division of Neurosurgery, Department of Surgery, Taichung Army Force General Hospital, Taichung 41152, Taiwan; sur060@gmail.com; 2 Department of Surgery, National Defense Medical Center, Taipei 11490, Taiwan; 3 General Education Center, College of Humanities and General Education, Central Taiwan University of Science and Technology, Taichung 406053, Taiwan; 4 Department of Laboratory Medicine, China Medical University Hospital, Taichung 404394, Taiwan; t6719@mail.cmuh.org.tw; 5 Graduate Institute of Biomedical Sciences, China Medical University, Taichung 404333, Taiwan; artbau2@gmail.com; 6 Department of Biomedical Imaging and Radiological Science, China Medical University, Taichung 404333, Taiwan; chyao@mail.cmu.edu.tw; 7 Department of Medical Laboratory Science and Biotechnology, China Medical University, Taichung 404333, Taiwan; home00274@gmail.com; 8 Department of Nursing, St. Mary’s Junior College of Medicine, Nursing and Management, Yilan 26644, Taiwan; yangjiunlong@gmail.com; 9 Department of Medical Laboratory Science and Biotechnology, College of Medical and Health Science, Asia University, Taichung 413305, Taiwan

**Keywords:** bioenergetic metabolism, KRAS, *let-7g*, MCPIP1, naringenin

## Abstract

The aberrant activation of signaling pathways contributes to cancer cells with metabolic reprogramming. Thus, targeting signaling modulators is considered a potential therapeutic strategy for cancer. Subcellular fractionation, coimmunoprecipitation, biochemical analysis, and gene manipulation experiments revealed that decreasing the interaction of kirsten rat sarcoma viral oncogene homolog (KRAS) with p110α in lipid rafts with the use of naringenin (NGN), a citrus flavonoid, causes lipid raft-associated phosphatidylinositol 3-kinase (PI3K)−GTP-ras-related C3 botulinum toxin substrate 1 (Rac1)−protein kinase B (Akt)-regulated metabolic dysfunction of glycolysis and mitochondrial oxidative phosphorylation (OXPHOS), leading to apoptosis in human nasopharyngeal carcinoma (NPC) cells. The use of *lethal-7g* (*let-7g*) mimic and *let-7g* inhibitor confirmed that elevated *let-7g* resulted in a decrease in KRAS expression, which attenuated the PI3K−Rac1−Akt−BCL-2/BCL-x_L_-modulated mitochondrial energy metabolic functions. Increased *let-7g* depends on the suppression of the RNA-specificity of monocyte chemoattractant protein-induced protein-1 (MCPIP1) ribonuclease since NGN specifically blocks the degradation of pre-let-7g by NPC cell-derived immunoprecipitated MCPIP1. Converging lines of evidence indicate that the inhibition of MCPIP1 by NGN leads to *let-7g* upregulation, suppressing oncogenic KRAS-modulated PI3K–Rac1–Akt signaling and thereby impeding the metabolic activities of aerobic glycolysis and mitochondrial OXPHOS.

## 1. Introduction

Metabolic reprogramming toward aerobic glycolysis has been considered a hallmark of cancer cells [1]. The different cancer cells utilize glycolysis and mitochondrial oxidative phosphorylation (OXPHOS) to supply the energy needed for rapid cell division, growth, invasion, metastasis, and resistance to apoptotic death triggered by extracellular matrix detachment or chemotherapeutic agents [2,3,4,5]. The metabolic adaptations of cancer cells critically influence the progression and response of cells to therapy [6]. Oncogenic regulator-governed signaling pathways are associated with the bioenergetics of the mitochondria of cancer cells [7]. Accumulating evidence supports the involvement of microRNAs (miRNAs) in modulating the bioenergetic metabolisms of cancer cells through the post-transcriptional regulation of aerobic glycolysis or mitochondrial OXPHO-related genes [8,9]. Among the miRNAs, *lethal-7* (*let-7*) has emerged as an essential regulator of glucose metabolic pathways in cancer cells [10,11]. *Let-7g* was downregulated and associated with nasopharyngeal carcinoma (NPC) progression [12]. Hepatocellular carcinoma cells with a high expression of B-cell lymphoma-extra large (BCL-x_L_) have attributed to a low *let-7g* expression [13]. In addition, *let-7g* exerts tumor-suppressive effect by imperfectly base-pairing with the 3′-untranslated region (UTR) of kirsten rat sarcoma viral oncogene homolog (*KRAS*) mRNA to regulate the oncogenesis of cancer cells [14,15]. The mRNA and protein expression levels of KRAS are upregulating in NPC tissues [16,17]. By interacting with the p110α catalytic subunit of phosphoinositide 3-kinase (PI3K), KRAS can cause constitutive activation of oncogenic PI3K–protein kinase B (Akt) signaling and promote the progression of lung cancer [18]. PI3K activation can result in the formation of active GTP-binding Ras-related C3 botulinum toxin substrate (GTP-Rac1) through a physical interaction between the p85α regulatory subunit of PI3K and Rac1, which enhances Akt activity and thereby induces the oncogenic transformation [19,20]. The inappropriate activation of PI3K–Rac1–Akt can rewire cell metabolism in a way that favors cell growth toward oncogenic development [21,22,23]. The functions of this pathway in coordinating the glycolytic metabolism of cancer cells are to command glucose transporter-1 (GLUT-1)-mediated glucose uptake, metabolic enzyme activities [23], and BCL-2/BCL-x_L_-dictated mitochondrial compartment biogenesis [23,24]. Thus, targeting the synergistic regulators of the KRAS–PI3K–Akt–Rac1 pathway would allow the dramatic attenuation of cancer cell metabolism.

A naturally occurring flavonoid compound, 2,3-dihydro-5,7-dihydroxy-2-(4-hydroxyphenyl)-4*H*-1-benzopyran-4-one, 4′,5,7-trihydroxyflavanone (naringenin, NGN), is predominantly found in grapes and citrus fruits [25]. It has been shown to inhibit cell growth in several human cancer cell lines through the downregulation of the PI3K–Akt signaling pathway [26,27]. NGN also exerts an inhibitory effect on glucose production in rat hepatoma cells [28]. PI3K–Akt signaling is prominently activated and dictates ER–mitochondria-mediated bioenergetics metabolism in NPC cells [29,30]. Despite the potential suppressive effect of NGN on the inhibition of cancer cell growth by attenuating the PI3K–Akt pathway, the mechanistic relatedness of NGN in suppressing World Health Organization (WHO) type 1 NPC (keratinizing subtype) cell metabolism remains understood. In three types of NPC, the overall survival rate and prognosis of type 1 are poor, even when radiation therapy is used [31,32]. Thus, this study explored the inactivation mechanism of PI3K–Akt–Rac1 by NGN, resulting in the metabolic attenuation of type 1 NPC cells.

## 2. Materials and Methods

### 2.1. Cell Culture

Epstein–Barr virus-negative human nasopharyngeal carcinoma (NPC) cell lines (NPC-TW 039 and NPC-TW 076) with a G→C mutation at codon 280 causing an arginine to threonine amino acid change were derived from a 64-year-old male patient and a 36-year-old female Chinese patient with keratinizing squamous cell carcinoma (WHO type I), respectively [33]. Both cell lines were obtained as previously described [34]. Smulow–Glickman (S-G) gingival epithelial cells were originally derived from normal adult human gingiva [35]. The cell lines were cultured in Dulbecco’s Modified Minimum Essential Medium (DMEM) (Gibco BRL, Grand Island, NY, USA) supplemented with 5% FBS and grown in 10 cm tissue culture dishes at 37 °C in a humidified incubator containing 5% CO_2_.

### 2.2. Chemicals, Reagents, and Plasmids

Bismaleimidohexane (BMH), Brij 98, chelerythrine (CHE), crystal violet, decyltriphenylphosphonium bromide (DecylTPP), 2-deoxyglucose (2-DG), dimethyl malonate (DMM), glucose, mitoquinone (MitoQ), myxothiazol (Myx), naringenin (NGN), oligomycin-A (Oligo-A), propidium iodide (PI), p-trifluoromethoxy carbonyl cyanide phenylhydrazone, rotenone (Rot), Tris-HCl, Triton X-100, and 3-(4,5-dimethylthiazol-2-yl)-2,5-diphenyltetrazolium bromide (MTT) were obtained from Sigma–Aldrich (St. Louis, MO, USA). The purity of NGN by high performance liquid chromatography (HPLC) analysis was >95.00%. The inhibitor of pan-caspase (Z-VAD-FMK) was purchased from Calbiochem (San Diego, CA, USA) and dissolved in dimethyl sulfoxide (DMSO). DMSO, potassium phosphate, negative mimic control miRNA, *let-7g* mimic, negative control miRNA inhibitor, and *let-7g* inhibitor were purchased from Merck (Darmstadt, Germany). Lipofectamine 2000 was obtained from Invitrogen (Carlsbad, CA, USA). DMEM, FBS, penicillin–streptomycin, trypsin–ethylenediaminetetraacetic acid (EDTA), and glutamine were received from Gibco BRL (Grand Island, NY, USA). The biotinylated annexin V was obtained from Thermo Fisher Scientific (New York, NY, USA). Centricon YM-100 was obtained from Millipore. pGFP short hairpin RNA (shRNA), pkirsten rat sarcoma viral oncogene homolog (KRAS) shRNAs, phemagglutinin (HA)-KRAS, and pFLAG-MCPIP1 were obtained from Addgene (Cambridge, MA, USA). Monocyte chemoattractant protein-induced protein-1 (MCPIP1) shRNA plasmid and Western blotting luminol reagent were purchased from Santa Cruz Biotechnology (Santa Cruz, CA, USA). D141N mutation was performed by amplifying the gene from pFLAG-MCPIP1 followed by the QuickChange site-directed mutagenesis method [36]. Recombinant MCPIP1 was obtained from Abcam (Cambridge, MA, USA). *Pre-let-7g* and *Pre-let-7f* were synthesized from Creative Biolabs (New York, NY, USA) according to the previously reported sequences [37]. The primer sequences were as follows: OCT-1, 5′-CCCTGTCTCAGCCCATACAGA-3′ and 5′-GCTGCAAATTGGTGGTTGGAT-3′; mtDNA, 5′-CGA AAGGACAAGAGA AATAAGG-3′, and 5′-CTGTAA AGTTTTAAGTTTTATGCG-3′; β-actin, 5′-GCTTGACTCAGGATTTAAAAACTGGAACGG-3′, and 5′-TATTCAACTGGTCTCAAGTCAGTGTACAGG-3′.

### 2.3. Antibodies

Anti-p85α, -protein kinase B (Akt), -phospho (p)-Akt (Ser 473), pro-apoptotic Bcl-2-associated x protein (BAX), Bcl-2 antagonist killer 1 (BAK), and B cell lymphoma 2 (BCL-2), antibodies were purchased from BD PharMingen. Antibodies directed against KRAS, p-BCL-2 (Ser 87), Bid, caspase-3, calnexin, p-cyclin dependent kinase 1 (CDK1) (Thr 161), glucose transporter-1 (GLUT-1), hexokinase II (HK-II), hypoxia-inducible factor 1α (HIF-1α), myeloid cell leukemia-1 (MCL-1), MCPIP1, pyruvate kinase type M2 (PKM2), pyruvate dehydrogenase kinase 1 (PDK1), and succinate dehydrogenase (SDH) were obtained from Abcam (Cambridge, MA, USA). Antibody against cytochrome *c* oxidase subunit II (Cox-2) was provided by Abbexa (Cambridge, UK). Anti-caspase-9, caspase-12, and caveolin-1 antibodies were received from Cell Signaling Technologies (Boston, MA, USA). Antibodies against CD55, CD71, CDK1, cyclin B1, p110α, and Ras-related C3 botulinum toxin substrate 1 (Rac1) were purchased from Santa Cruz Biotechnology. Anti-B-cell lymphoma-extra large (BCL-x_L_), pan-cadherin, anti-phosphatase and tensin homologs deleted from chromosome 10 complexes (PTEN), and anti-p-PTEN (Ser 380/Thr 382/Ser 385) antibodies were obtained from Thermo Fisher Scientific (New York, NY, USA). Antibodies against β-actin, p-BCL-2 (Thr 69), p-BCL-x_L_ (Ser 62), hemagglutinin (HA)-epitope tag, and α-tubulin were obtained from Sigma–Aldrich. Horseradish peroxidase (HRP)-conjugated streptavidin was provided by Thermo Fisher Scientific (New York, NY, USA). HRP-conjugated anti-mouse, -goat, and -rabbit IgG secondary antibodies were purchased from Jackson ImmunoResearch Laboratory (West Grove, PA, USA). Detailed information regarding the source and concentration of antibodies used in this study is presented in Supplementary Table S1.

### 2.4. Measurement of DNA Fragmentation

Histone-associated DNA fragments were assayed using the cell death detection enzyme-linked immunosorbent assay (ELISA) kit (Roche Applied Science, Mannheim, Germany). Briefly, cells (5 × 10^4^) were incubated in a hypertonic buffer for 30 min at room temperature. After centrifugation, the cell lysates were transferred to an anti-histone-coated microplate to bind histone-associated DNA fragments. The plates were washed after 1.5 h of incubation, and nonspecific binding sites were saturated with blocking buffer. The plates were then incubated with peroxidase-conjugated anti-DNA for 1.5 h at room temperature. To determine the amount of retained peroxidase, 2,2’-azino-di-(3-ethylbenzthiazoline-6-sulfonate) was added as a substrate, and a spectrophotometer (Thermo Labsystems Multiskan Spectrum, Franklin, MA, USA) was used to measure the absorbance at 405 nm [30].

### 2.5. Rac1 Activation Assay

Treated cells or caveolin-1 and CD55-riched DRM fractions prepared from treated cells were lysed by incubation with Rac1 lysis buffer (50 mM Tris-HCl (pH 7.4), 100 mM NaCl, 1 mM MgCl_2_, 20 mM β-glycerophosphate (pH 7.5), 1% NP-40, 10% glycerol, 10 mM NaF, 2 mM Na_3_VO_4_, 5 mM dithiothreitol, 0.5 mM phenylmethylsulfonyl fluoride, 1 μg/mL leupeptin, and 1 μg/mL pepstatin) for 15 min at 4 °C. The lysates were centrifuged at 14,000× *g* for 10 min in a microcentrifuge at 4 °C. The lysates and immunoprecipitated complexes were incubated with 40 μg of bacterially expressed glutathione-S-transferase (GST)-PAK-CD fusion protein prebound to glutathione agarose beads for 30 min at 4 °C. The beads were pelleted, washed with 500 μL of Rac1 lysis buffer, mixed with 1× SDS sample buffer (50 mM Tris-HCl (pH 6.8), 2% SDS, 0.1% bromophenol blue, 10% glycerol, and 100 mM dithiothreitol), and incubated for 5 min at 100 °C. The samples were then separated by 10% SDS-PAGE and immunoblotting with an antibody against Rac1 [30].

### 2.6. Measurement of the Oxygen Consumption Rate (OCR) and Extracellular Acidification Rate (ECAR)

The OCR and ECAR were determined by using the Seahorse XFe96 Extracellular Flux Analyzer (Agilent, CA) as previously described [38]. Briefly, cells were seeded in XF96 Cell Culture Microplates and incubated at 37 °C for 24 h. Then, the culture medium was changed to XF assay medium supplemented with 1 mM pyruvate, 2 mM glutamine, and 10 mM glucose for OCR assay or 1 mM glutamine for ECAR assay and placed in a 37 °C incubator without CO_2_ for an hour. The OCR was measured in the presence of 1 μM mitochondrial F_0_F_1_-ATPase inhibitor Oligo-A, 0.5 μM of the oxidative phosphorylation uncoupler FCCP, and the respiratory chain inhibitor Rot (30 μM) plus mitochondrial complex III inhibitor Myx (10 μM) at the indicated time points. ECAR was examined under basal conditions and during the sequential addition of glucose, 1 μM Oligo-A, and glycolysis inhibitor 2-DG (25 mM) at the indicated time points. The OCR and ECAR were measured using a Seahorse Bioscience XF24 Analyzer. After each assay, cells were lysed, and the protein concentration was measured to normalize the OCR and ECAR.

### 2.7. Glucose Uptake Assay

Cell glucose uptake was measured using Glucose Uptake-Glo Assay (Promega, Madison, MI, USA). Briefly, cells were plated in 96-well plates at a density of 1 × 10^4^ cells per well to allow for attachment overnight. After 36 h, the cells were treated with 500 μM 2-deoxyglucose (2-DG, 50 μL/well) for 20 min at room temperature. Luciferase activities were measured at the end of the glucose uptake on a microplate luminometer. The rate of glucose uptake was analyzed according to the manufacturer’s instructions.

### 2.8. Measurement of Cellular ATP

The cellular ATP content was measured by the ATP-based CellTiter-Glo Luminescent Cell Viability kit (Promega, Madison, MI, USA) with modifications from the manufacturer’s protocol. Briefly, cells were plated in 24-well plates at 2 × 10^4^ cells per well to allow for attachment overnight. The cells were grown to ~60% confluence and treated with vehicle or NGN for 36 h. At the end of the incubation, an equal volume of the single one-step reagent provided in the kit was added to each well and blocked for 15 min at room temperature. The cellular ATP content was measured by a luminescent plate reader. An additional plate with the same setup was used for cell counting by hemocytometer to normalize the cell number for calculating the ATP level [30].

### 2.9. Measurement of Cellular Pyruvate and Lactate

Pyruvate and lactate levels were determined by the Pyruvate Assay Kit and Lactate Assay Kit (Sigma–Aldrich, St. Louis, MO, USA), respectively. Briefly, cells were plated in 96-well plates at a density of 1 × 10^4^ cells per well to allow for attachment overnight. After 36 h, the cells were lysed in 200 µL Lactate Assay Buffer or Lactate Assay Buffer and centrifuged at 13,000× *g* for 10 min. Cell supernatants were centrifuged twice at 2000× *g* for 5 min to remove cells debris. The samples were deproteinized with a Micronco-10 kDa MWCO Centrifugal Filter (Merck Millipore, MA, USA). Pyruvate and lactate concentrations were read using a Synergy HT (Bio-TEK, Winooski, VT) plate reader. Values were normalized to cellular protein concentrations.

### 2.10. Measurement of Mitochondrial ROS Production

As previously described, mitochondrial ROS was measured by the MitoSOX-based flow cytometric assay (Invitrogen, Carlsbad, CA, USA) [39]. Briefly, 2 × 10^4^ cells were incubated with 2 μM MitoSOX Red superoxide indicator for 30 min and washed, and the cells were then analyzed on a FACSCount flow cytometer.

### 2.11. Measurement of Intracellular Glucose

The intracellular glucose of cells was measured using a Glucose Assay Kit (Abcam, Cambridge, MA, USA) according to the manufacturer’s instructions. Briefly, cells were plated in 24-well plates at a density of 2 × 10^4^ cells per well to allow overnight attachment. The cells were grown to ≈60% confluence and treated with vehicle or NGN for the indicated periods. At the end of the incubation, cell lysates of 1 μL were added to 96-well plates. The volume was adjusted to 50 μL/well with Lactate Assay Buffer before the addition of 50 μL Glucose Reaction Mix (composed of 46 μL Glucose Assay Buffer, 1 μL Glucose Enzyme Mix, and 1 μL Glucose Probe) to each well and incubation at room temperature in the dark for 10 min. The absorbance was determined using a microplate reader (EL340 Bio-TEK Instruments, Winooski, VT, USA) at 570 nm. The glucose concentration was derived from the absorbance using a standard curve. Glucose levels were normalized to protein levels [30].

### 2.12. Measurement of Cytosolic Calcium (Ca^++^)

The Ca^++^ level was determined by measuring the retention of Indo-1/AM. Briefly, the treated cells (5 × 10^4^) were incubated with 3 μg/mL Indo-1/AM for 30 min at 37 °C. The cells were then pelleted by centrifugation at 160× *g*. The pellets were resuspended and washed twice with PBS. The level of Ca^++^ was evaluated as previously described [30].

### 2.13. Determination of Let-7 Expression by Quantitative Real-Time PCR

RNA was extracted using TRIzol reagent (Invitrogen Life Technologies, Carlsbad, CA, USA) according to the manufacturer’s instructions. First-strand cDNA synthesis was performed using 1 μg of total RNA in the presence of Superscript II reverse transcriptase (Gibco BRL, Grand Island, NY, USA) and specific primers as reported previously [40,41]. The reverse transcription was carried out at 60 °C for 45 min and terminated by a further incubation at 85 °C for 5 min. Real-time PCR was performed on a LightCycler^®^ 1.5 Instrument (Roche Diagnostics, Basel, Switzerland) using the LightCycler^®^ TaqMan^®^ Master kit. The PCR mixture (25 μL) consisted of 5 μL cDNA template, 0.32 μM *let-7* gene-specific primers [40,41], and 2 μL LightCycler^®^ TaqMan^®^ Master Mix. The thermal cycling conditions consisted of incubation for 5 min at 95 °C followed by 40 denaturation–amplification cycles for 10 s at 95 °C, annealing for 20 s at 60 °C, and extension for 20 s at 72 °C. During the amplification process, the fluorescence emitted by the reporter dye was monitored by the threshold cycle (*Ct*). The *Ct* of each sample was recorded as a quantitative measure of the amount of PCR product in the sample. The data were normalized against the relative quantity of U6 and expressed as Δ*Ct* = (*Ct*_let-7_ − *Ct*_U6_). LightCycler^®^ software version 3.5 was used to automatically calculate the concentration of *let-7* copies in the experimental samples. The values were normalized to *U6*. The results were obtained from three independent experiments.

### 2.14. In Vitro RNA Cleavage Assay

The assay was performed according to previously reported methods [42]. The immunoprecipitate MCPIP1 or recombinant MCPIP1 (5 pmol) were mixed with one pmol of *pre-let-7g* or *pre-let-7f* in the presence of vehicle (−) or the indicated concentrations of NGN and incubated at 37 ° C for 60 min in reaction buffer (50 mM Tris, 150 mM NaCl, 2.5 mM MgCl_2_, 2.5 mM DTT, 0.5 mM EDTA, 0.025 mM ZnCl_2_, pH 8.3). After the reaction, samples were denatured for 15 min at 70 °C, analyzed by 4% SDS-PAGE in 1× Tris-borate-EDTA (TBE) buffer, and then subjected to Northern blot analysis.

### 2.15. Pyruvate Kinase Type M2 (PKM2) Activity Assay

The activity of PKM2 was measured according to previously reported methods [43]. Briefly, a reaction was initiated by the addition of the PKM2 proteins immunoprecipitated from the treated or transfected whole cell lysates into the reaction buffer (30 mM pyruvate, 6.6 mM NADH, 200 mM Tris-HCl, pH 7.3) in the presence of Fructose-1,6-Biphosphate (500 mM) for 30 min at room temperature. PKM2 activity was then measured with a colorimetric-based pyruvate kinase activity assay kit (BioVision, Milpitas, CA, USA) according to the manufacturer’s protocol.

### 2.16. Pyruvate Dehydrogenase Kinase 1 (PDK1) Activity Assay

The enzymatic activity of PDK1 was determined using the ADP-GloTM Kinase Assay Kit (Promega, Madison, MI, USA). Briefly, PDK1 proteins immunoprecipitated from the treated or transfected whole cell lysates were incubated with kinase buffer (50 mM Hepes, pH 7.4, 1 mM dithiothreitol, and 10 mM MgCl_2_) in the presence of 5 μM ATP and 0.2 μg/μL PDKtide (KTFCGTPEYLAPEVRREPRILSEEEQEMFRDFDYIADWC) substrate for 60 min at room temperature. The reaction was stopped by the addition of 5 μL of ADP-Glo reagent followed by incubation for 40 min at room temperature. PDK1 activity was then measured as the luminescence-based pyruvate kinase activity according to the manufacturer’s protocol.

### 2.17. Hexokinase II (HK-II) Activity Assay

The HK-II activity was determined using Colorimetric Hexokinase Activity Assay (Abcam, Cambridge, MA, USA). Briefly, HK-II proteins immunoprecipitated from the treated or transfected whole cell lysates were incubated with the Reaction Mix (assay buffer, developer, coenzyme, and hexokinase substrate) for 30 min at room temperature protected from light. HK-II activity was then measured as the colorimetric-based pyruvate kinase activity according to the manufacturer’s protocol.

### 2.18. Lactate Dehydrogenase (LDH) Activity Assay

The LDH activity was determined using Colorimetric Hexokinase Activity Assay (Abcam, Cambridge, MA, USA). Briefly, LDH proteins immunoprecipitated from the treated or transfected whole cell lysates were incubated with Master Reaction Mix (LDH assay buffer and LDH substrate) for 30 min at room temperature. LDH activity was then measured as the colorimetric-based pyruvate kinase activity according to the manufacturer’s protocol.

### 2.19. Succinate Dehydrogenase (SDH) Activity Assay

Mitochondria were isolated according to the protocol previously described [30]. The SDH activity was determined using the Succinate Dehydrogenase Activity Colorimetric Assay Kit (Sigma–Aldrich Inc., St. Louis, MO, USA). It was measured by monitoring the decrease in absorbance at 600 nm for 10 min in the presence of 0.1 M sodium succinate (pH 7.5), 0.05 mM 2,6-dichlorophenolindophenol, 0.1 M NaPO_4_ buffer (pH 7), and 50–100 μg of resuspended biological membranes in a 1 mL reaction volume. SDH activity was expressed relative to the background reduction of 2,6-dichlorophenolindophenol (membranes without succinate).

### 2.20. Determination of Succinate

According to the manufacturer’s instructions, the succinate level was determined by the Succinate Colorimetric Assay Kit (Sigma–Aldrich Inc., St Louis, MO, USA). Briefly, cell lysates (50 μg) were homogenized on ice in 500 μL of ice-cold Succinate Assay Buffer and centrifuged at 10,000× *g* for 5 min to remove cell debris. Then, the samples were added to a 96-well plate and mixed with the appropriate reaction mix. The resultant mixtures were further incubated at 37 °C for 30 min. The amount of succinate was determined by using a spectrophotometer (Thermo Labsystems Multiskan Spectrum, Franklin, MA, USA) at 450 nm.

### 2.21. Mitochondrial DNA (mtDNA) Copy Number

DNA was extracted from treated cells using the QIAamp DNA Mini Kit (Qiagen, Hilden, Germany) according to the manufacturer’s instructions. The PCR reaction mixture (25 μL) consisted of 6 ng of mtDNA template, 0.4 μM of mtDNA target specific primer pair, 0.15 μM of TaqMan hydrolysis probe, and 2 μL of LightCycler^®^ TaqMan^®^ Master. The thermal cycling conditions consisted of incubation for 10 min at 95 °C followed by 40 denaturation amplification cycles for 15 s at 95 °C, annealing for 60 s at 50 °C, and extension for 1 sec at 72 °C. During the amplification process, fluorescence emitted by the reporter dye was monitored by the threshold cycle (*Ct*). The *Ct* of each sample was recorded as a quantitative measure of the amount of PCR product in the sample. The data were normalized against the relative quantity of β-actin and expressed as △*Ct* = (*Ct*_mtDNA_ − *Ct*_β-actin DNA_).

### 2.22. Statistical Analysis

Statistical analyses of the data were performed using the unpaired Student’s *t*-test and one-way ANOVA; *p* < 0.05 was considered statistically significant.

## 3. Results

### 3.1. The Impairment of the Regulation of Glycolysis and Mitochondrial Oxidative Phosphorylation (OXPHOS) Confers the Apoptotic Death of NPC Cells

**Figure 1:**
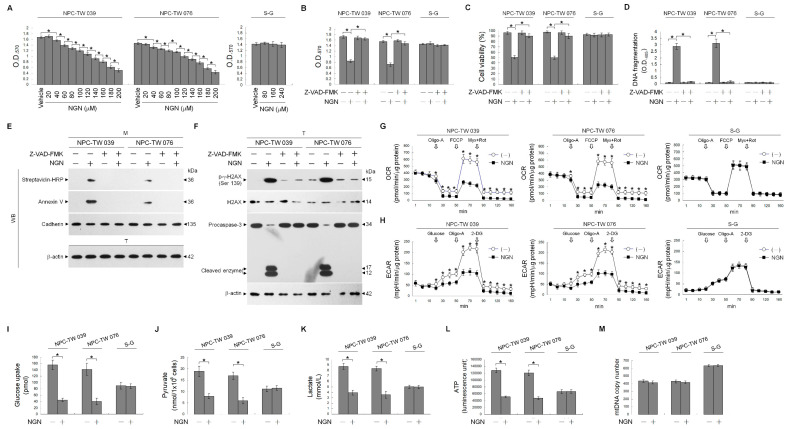
Dysfunction of glycolysis and mitochondrial oxidative phosphorylation (OXPHOS) by naringenin (NGN) confers apoptosis in nasopharyngeal carcinoma (NPC) cells. (**A**) The effect of NGN on NPC and Smulow–Glickman (S-G) cell growth. Cells treated with vehicle (−) or the indicated concentrations of NGN for 36 h. The 3-(4,5-dimethylthiazol-2-yl)-2,5-diphenyltetrazolium bromide (MTT) assay determined cell growth. (**B**–**F**) The effects of NGN on the induction of NPC cell growth inhibition and apoptosis. After 36 h treatment with (−), NGN (160 μM), Z-VAD-FMK (8 μM) or NGN (160 μM) and Z-VAD-FMK (8 μM), cell growth and viability were determined by the MTT and flow cytometric analysis of PI uptake, respectively. DNA fragmentation was determined using a Cell Death Detection enzyme-linked immunosorbent assay (ELISA) kit. Annexin V-biotinylated vehicle- or NGN-treated cells were fractionated by subcellular fractionation centrifugation to isolate the plasma membrane (M) fraction. The levels of the indicated proteins in the lysates of (−)-, NGN-, -Z-VAD-FMK, and the NGN plus Z-VAD-FMK co-treated M fraction were determined by Western blot analysis using streptavidin-horseradish peroxidase (HRP) and specific antibody to Annexin V or cadherin. Antibody against cadherin was used as an internal control for the plasma membrane. The levels of phosphorylated histone H2A.X (Ser 139) (p-γ-H2AX (Ser 139)), H2AX, and caspase-3 in the total cell (T) lysates were determined by Western blot analysis with specific antibodies. β-Actin was used as an internal control for sample loading. (**G**,**H**) The effects of NGN on glycolysis and mitochondrial oxidative phosphorylation (OXPHOS). Cells treated with vehicle or NGN (160 μM) for the indicated periods. The oxygen consumption rate (OCR) was measured in the presence of oligomycin-A (Oligo-A) (1 μM), carbonylcyanide-p-trifluoromethoxyphenylhydrazone (FCCP) (0.5 μM), and rotenone (Rot) (30 μM) plus myxothiazol (Myx) (10 μM) at the indicated time points. The extracellular acidification rate (ECAR) was examined under the sequential addition of glucose, Oligo-A (1 μM), and 2-deoxy-D-glucose (2-DG) (25 mM) at the indicated time points. The OCR and ECAR were measured using a Seahorse Bioscience XF24 Analyzer. (**I**–**M**) The effects of NGN on the levels of glucose uptake, pyruvate, lactate, ATP, and mitochondrial DNA (mtDNA) copy number. After 36 h of treatment with (−) or NGN (160 μM), the pyruvate, lactate, and ATP values were analyzed using the Pyruvate Assay kit, Lactate Assay kit, and ATP-based CellTiter-Glo Luminescent Cell Viability kit, respectively. Glucose uptake was measured using the Glucose Uptake Colorimetric assay kit. The level of mtDNA was analyzed using quantitative real-time PCR (qRT-PCR). The mtDNA expression was determined relative to that of β-actin. The values are presented as three independent experiments’ mean ± standard error. * *p* < 0.05: significantly different from vehicle- or NGN-treated cells.

To assess whether sex differences affect cell sensitivity to naringenin (NGN), two human NPC-TW 039 and NPC-TW 076 cell lines derived from one male and one female Chinese patient with WHO type 1 keratinizing squamous cell carcinoma were used in the study. Figure 1A shows that NGN dose-dependently inhibited NPC cell proliferation with a half-maximal inhibitory concentration (IC_50_) of 160 μM. The growth of normal human gingival epithelial S-G cells was not affected by 160 μM NGN, even at concentrations of 240 μM (Figure 1A). The IC_50_ value of NGN with apparent cytotoxicity of NPC cells (but not of S-G cells) could be validated by flow cytometry analysis of propidium iodide (PI)-stained cells (Figure 1C). NPC cells dying by apoptosis was verified by observation of Annexin-V binding, DNA fragmentation, DNA double-strand break marker histone H2A.X (Ser 139) phosphorylation, and procaspase-3 cleavage when treated with 160 μM NGN (Figure 1D–F). NGN-induced growth-suppressing and apoptotic effects were inhibited by the broad-spectrum caspase inhibitor Z-VAD-FMK (Figure 1B–F). All subsequent experiments used a concentration of 160 μM to treat cells. The oxygen consumption rate (OCR), an indicator of mitochondrial respiration, was significantly reduced by treatment with the mitochondrial F_0_F_1_-ATPase inhibitor oligomycin-A (Oligo-A). After the addition of an oxidative phosphorylation uncoupler (carbonylcyanide-p-trifluoromethoxyphenylhydrazone, FCCP), the OCR was reversed and achieved the maximum level. Subsequently, a decline in the OCR could be observed upon the incorporation of respiratory chain inhibitor rotenone (Rot) and mitochondrial complex III inhibitor myxothiazol (Myx) (Figure 1G). The extracellular acidification rate (ECAR), an indicator of net proton loss during glycolysis, reached its maximum level after glucose treatment and the addition of oligonucleotide-A. Treatment with glycolysis inhibitor 2-deoxy-D-glucose (2-DG) reduced the ECAR to the basal level (Figure 1H). These data indicated that NPC and S-G cells underwent mitochondrial OXPHOS and glycolysis for energy generation. Biochemical analysis showed that NPC and S-G cells took up glucose and generated end products (pyruvate and lactate) of aerobic glycolytic metabolism (Figure 1I–K). Adenosine triphosphate (ATP) synthesis efficiency was detected (Figure 1L). In contrast to the lack of an impact of NGN on S-G cells, NGN exerted inhibitory effects on mitochondrial OCR, ECAR, glucose uptake, pyruvate, lactate, and ATP levels in NPC cells; however, the copy number of mitochondrial DNA (mtDNA) did not decrease during NGN treatment (Figure 1M). These results indicate that the apoptosis induction of NPC cells by NGN may result from the impaired regulation of aerobic glycolysis and mitochondrial OXPHOS and not from the altered mtDNA copy number. 

### 3.2. Decreased Interaction of KRAS with p110α by NGN Confers the Suppression of Lipid Raft-Associated KRAS–PI3K–Rac1–Akt-Mediated Bioenergetic Generation

**Figure 2:**
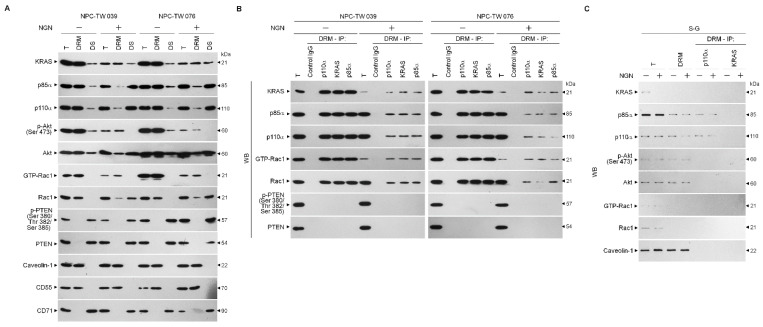
Decreased level of kirsten rat sarcoma viral oncogene homolog (KRAS) in the lipid rafts causes attenuation in the formation of lipid raft-associated KRAS–phosphatidylinositol 3-kinase (PI3K)–active GTP-binding Ras-related C3 botulinum toxin substrate (GTP-Rac1) complexes. (**A**) Nasopharyngeal carcinoma (NPC) cells were treated with vehicle (−) or naringenin (NGN) for 36 h. Detergent-resistant membrane (DRM) and detergent-soluble (DS) fractions were prepared by flotation along a sucrose density gradient. The levels of the indicated proteins in the lysates of (−)- or NGN-treated DRM and DS fractions and total cell (T) lysates were determined by Western blot analysis using specific antibodies. Antibodies against caveolin-1/CD55 and CD71 were used as internal controls for DRM and DS fractions. (**B**,**C**) After 36 h of treatment with (−) or NGN, DRM fractions were prepared by flotation along a sucrose density gradient. The antibody used for coimmunoprecipitation is indicated at the top. The proteins from the immunoprecipitated complexes were detected using Western blotting with specific antibodies. Normal IgG was used as a control for antibody specificity. Total and DRM lysates from (−)- or NGN-treated cells were used to monitor the indicated protein levels and were determined using Western blot analysis with specific antibodies.

The spatial compartmentalization of effector molecules in the lipid rafts is essential for activating the specialized signaling pathway that confers the bioenergetics of cancer cells [44]. Thus, we wondered whether kirsten rat sarcoma viral oncogene homolog (KRAS)–phosphatidylinositol 3-kinase (PI3K)–Ras-related C3 botulinum toxin substrate 1 (Rac1)–protein kinase B (Akt) signaling-regulated molecules in the lipid rafts were affected by NGN. Lipid rafts, detergent-resistant membranes (DRMs) [45,46], were isolated by sucrose gradient centrifugation and clarified by the presence of caveolin-1 and CD55. The NGN treatment reduced the KRAS levels in NPC cell DRMs, which was attributed to a decrease in expression, as the results showed lower levels of KRAS detected in the total cell lysate compared to vehicle treatment. Decreased p85α and p110α in the DRM fractions occurred with increased levels of p85α and p110α in the DS fractions. In the meantime, phospho (p)-Ak) (Ser 473) and active Rac1 (GTP-bound Rac1; GTP-Rac1) decreased in the DRM fractions with a concomitant increase in unphosphorylated Akt and inactive Rac1 in the DR fractions. Phosphatase and tensin homologs deleted from chromosome 10 complexes (PTEN), a negative regulator of phosphoinositide 3-kinase (PI3K)-dependent Akt activation, showed no change in their localization in the DS; only the phosphatase-inactive form of p-PTEN (Ser 380/Thr 382/Ser 385) was detected in the DS fractions (Figure 2A). By contrast, KRAS was not detected in the DRM fractions of S-G cells. NGN treatment was unable to decrease the levels of p85α, p110α, p-Akt (Ser 473), and GTP-Rac1 but suppressed KRAS expression, and it failed to affect the localization of p85α, p110α, and p-Akt (Ser 473) in the DRMs (Figure 2C). These results suggest that decreasing KRAS levels in the lipid rafts were involved in the inactivation of PI3K–Rac1–Akt signaling in the NPC cells.

Since KRAS enhances PI3K–Akt-mediated signal transduction via interaction with p110α to serve as an oncogenic transducer of metabolism [18], we examined the physical interaction of KRAS, p85α, p110α, Rac1, or PTEN in the lipid rafts. Immunoprecipitation of DRM proteins derived from vehicle-treated NPC cells by p110α, KRAS, or p85α antibody and Western blot analysis revealed that KRAS, p85α, p110α, and GTP-Rac1 formed a complex in the lipid rafts. Immunoprecipitation of p110α by a specific antibody from the DRM fractions of NGN-treated NPC cells exhibited a lower KRAS and GTP-Rac1 interaction with the p85α:p110α immunoprecipitates. Re-immunoprecipitation with anti-KRAS or anti-p85α antibody confirmed that NGN treatment decreased KRAS and GTP-Rac1 coimmunoprecipitation in precipitated p85α:p110α (Figure 2B). A coimmunoprecipitation of DRM fractions of vehicle-treated S-G cells using an antibody specific for p110α or KRAS showed that p85α formed a complex with p110α but did not interact with KRAS, GTP-Rac1, and p-Akt (Ser 473). However, NGN treatment did not suppress the incorporation of p85α and p110α into the DRMs to form the p85α–p110α complexes (Figure 2C). These observations indicate that reducing the level of KRAS and its interaction with p110α in the lipid rafts caused the decline of PI3K-mediated Rac1 and Akt activation in the NPC cells.

**Figure 3:**
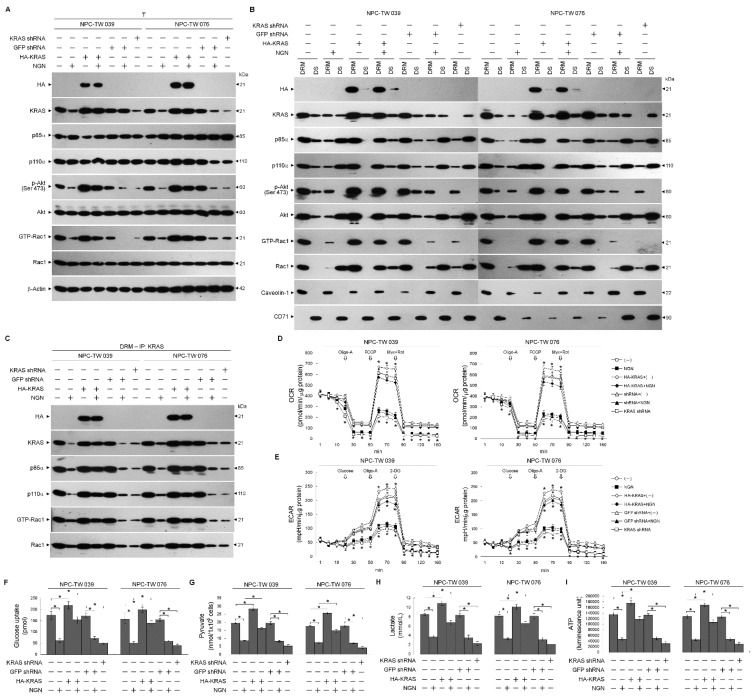
Disruption of kirsten rat sarcoma viral oncogene homolog (KRAS)–phosphatidylinositol 3-kinase (PI3K)–active GTP-binding Ras-related C3 botulinum toxin substrate (GTP-Rac1) complex formation and impaired energetic synthesis of the glycolysis and mitochondrial respiration pathways. (**A**–**C**) At 12 h after transfection with hemagglutinin (HA)-KRAS, GFP short hairpin RNA (shRNA), or KRAS shRNA, cells were treated with vehicle (−) or naringenin (NGN) for 36 h. The levels of the indicated proteins in the lysates of the total cell (T), detergent-resistant membrane (DRM), and detergent-soluble (DS) fractions were determined by Western blot analysis using specific antibodies. Co-immunoprecipitation of KRAS, p85α, p110α, and GTP-Rac1 was performed using the DRM fractions prepared from the cells treated as described above. The KRAS antibody used for co-immunoprecipitation is indicated at the top. The proteins from the immunoprecipitated complexes were detected using Western blotting with specific antibodies. Normal IgG was used as a control for antibody specificity. (**D**,**E**) Oxygen consumption rate (OCR) was measured in the presence of oligomycin-A (Oligo-A), carbonylcyanide-p-trifluoromethoxyphenylhydrazone (FCCP), and rotenone (Rot) plus myxothiazol (Myx) at the indicated time points. Extracellular acidification rate (ECAR) was examined in the sequential addition of glucose, Oligo-A, and 2-deoxy-D-glucose (2-DG) at the indicated time points. The OCR and ECAR were measured using a Seahorse Bioscience XF24 Analyzer. (**F**–**I**) The pyruvate, lactate, and ATP values were analyzed using the Pyruvate Assay kit, Lactate Assay kit, and ATP-based CellTiter-Glo Luminescent Cell Viability kit, respectively. In addition, glucose uptake was measured using the Glucose Uptake Colorimetric assay kit. The values are presented as three independent experiments’ mean ± standard error. * *p* < 0.05: significantly different from vehicle-treated empty vector-transfected, vehicle-treated GFP-transfected, or NGN-treated empty vector-transfected cells.

The action of NGN to decrease the ability of KRAS from interfering with the formation of the KRAS–p110α–p85α–GTP-Rac1 complexes in the lipid rafts promoted us to test whether the functions of glycolysis and mitochondrial OXPHOS were affected in the NPC cells. By employing a hemagglutinin (HA) epitope-tagged KRAS (HA-KRAS), elevated levels of KRAS–p110α–p85α–GTP-Rac1 complexes in the DRM fractions overcome the suppression of Akt (Ser 473) phosphorylation, Rac1 activation, and KRAS–p110α complex formation by NGN (Figure 3A–C). In addition, the impairment of glucose uptake, ECFR, mitochondrial OCR, pyruvate, lactate, and ATP generation was rescued by the overexpression of HA-KRAS (Figure 3D–I and Supplementary Tables S2.1 and S2.2). Similar to the NGN-treated cells, cells expressing KRAS short hairpin RNA (shRNA) resulted in decreased levels of lipid raft-associated KRAS–p110α–p85α–GTP-Rac1 complexes, KRAS, p-Akt (Ser 473), GTP-Rac1, glucose uptake, pyruvate, lactate, ATP, ECFR, and mitochondrial OCR compared with GFP shRNA-transfected cells (Figure 3A–I and Supplementary Tables S3.1 and S3.2). These results indicate that the suppression of bioenergetic generation by NGN occurs due to the attenuation of the formation of KRAS–PI3K–GTP-Rac1 complexes by reducing KRAS interaction with p110α.

### 3.3. NGN-Induced KRAS–PI3K–Rac1–Akt-Modulated Metabolic Dysfunction of Glycolysis and Mitochondrial OXPHOS Associated with the Up-Regulation of let-7g

**Figure 4:**
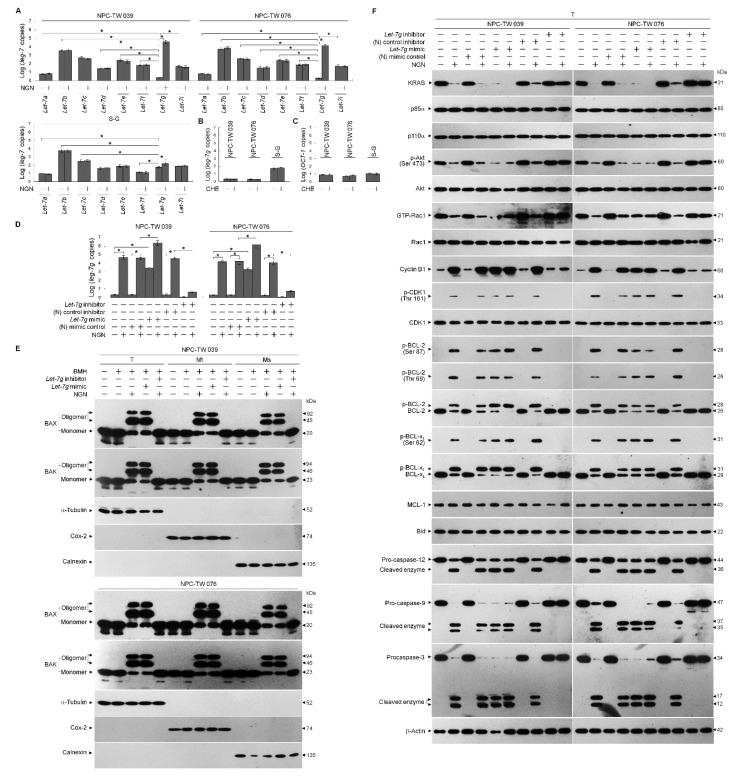

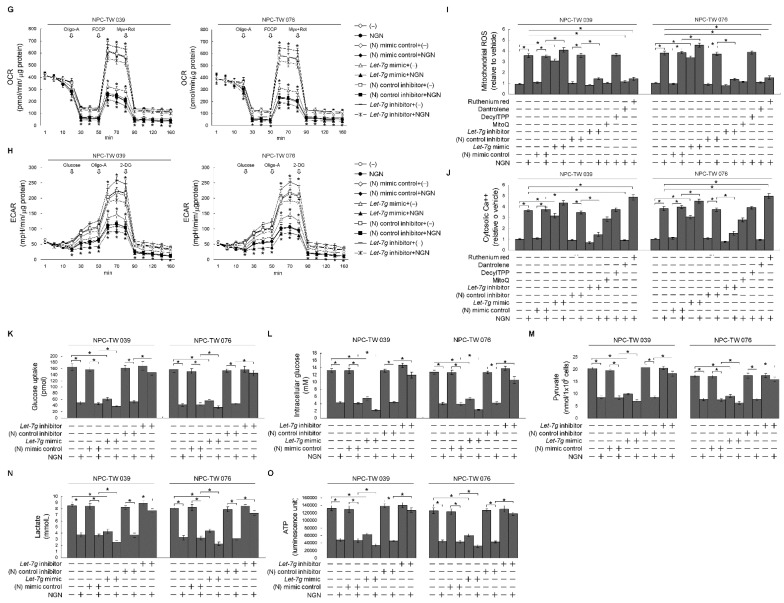
Metabolic dysfunction of glycolysis and mitochondrial oxidative phosphorylation associated with *lethal-7g* (*let-7g*)-attenuated kirsten rat sarcoma viral oncogene homolog (KRAS)–phosphatidylinositol 3-kinase (PI3K)–Ras-related C3 botulinum toxin substrate (Rac1)–protein kinase B (Akt) signaling. (**A**) Cells were treated with vehicle (−) or naringenin (NGN) for 36 h. The expression of *let-7* was determined using quantitative real-time polymerase chain reaction (qRT-PCR). The *let-7* value was normalized to the U6 level. The Y-axis shows the denary logarithm of the normalized *let-7* copy number. (**B**,**C**) The effects of the protein kinase C (PKC) inhibitor chelerythrine (CHE) on *let-7g* and *OCT-1* expression. After treating with CHE (0.5 μM) for 36 h, the relative expression levels of *let-7g* and *OCT-1* were determined by qRT-PCR. The *let-7g* and *OCT-1* values were normalized to the U6 level and β-actin, respectively. The Y-axis shows the denary logarithm of the normalized *let-7g* or *OCT-1* copy number. (**D**–**F**) At 12 h after transfection with the negative (N) mimic control, *let-7g* mimic, (N) mimic control inhibitor, or *let-7g* inhibitor, cells were treated with (−) or NGN for 36 h. Five mM bismaleimidohexane (BMH)-treated cells were subjected to subcellular fractionation to obtain the mitochondrial (Mt) and endoplasmic reticulum (ER)/microsomal (Ms) fractions. In total, 20 μg of total protein from the recovered fractions was analyzed by 10% sodium dodecyl sulfate polyacrylamide gel electrophoresis (SDS-PAGE) and probed with specific antibodies, as indicated. Cytochrome *c* oxidase subunit II (Cox-2), calnexin, and α-tubulin were used as internal controls for the mitochondria, ER, and cytosol, respectively. The levels of the indicated proteins in the total cell (T) lysates were determined by Western blot analysis using specific antibodies. (**G**,**H**) At 12 h after transfection with the (N) mimic control, *let-7g* mimic, (N) mimic control inhibitor, or *let-7g* inhibitor, cells were treated with (−) or NGN in the presence of 1 μM oligomycin-A (Oligo-A), carbonylcyanide-p-trifluoromethoxyphenylhydrazone (FCCP), and rotenone (Rot) plus myxothiazol (Myx) or the sequential addition of glucose, Oligo-A, and 2-DG at the indicated time points. The oxygen consumption rate (OCR) and extracellular acidification rate (ECAR) were measured using a Seahorse Bioscience XF24 Analyzer. (**I**,**J**) At 12 h after transfection with the (N) mimic control, *let-7g* mimic, (N) mimic control inhibitor, or *let-7g* inhibitor, cells were treated with vehicle (−), NGN, NGN plus Mitoquinone (MitoQ) (10 μM), decyltriphenylphosphonium bromide (DecylTPP) (1 μM), NGN plus dantrolene (25 μM), or NGN plus ruthenium red (1 μM) for 36 h. Flow cytometry determined the levels of mitochondrial reactive oxygen species (ROS) and cytosolic calcium (Ca^++^) by measuring the increased fluorescence. (**K**–**O**) Transfected cells were harvested 36 h after treatment with (−) or NGN. The glucose, pyruvate, lactate, and ATP values were analyzed using the Glucose assay, Pyruvate Assay kit, Lactate Assay kit, and ATP-based CellTiter-Glo Luminescent Cell Viability kit, respectively. In addition, glucose uptake was measured using the Glucose Uptake Colorimetric Assay kit. The values are presented as three independent experiments’ mean ± standard error. * *p* < 0.05: significantly different from vehicle-treated empty vector-transfected, vehicle-treated (N) mimic control-transfected, or vehicle-treated (N) mimic control inhibitor-transfected cells.

Given the role of *let-7g* in controlling tumorigenesis by targeting the *KRAS* 3′-untranslated region (UTR) to attenuate its expression [14,15], quantitative real-time polymerase chain reaction (qRT-PCR) was performed to analyze the expression levels of *let-7* family members. *Let-7g* displayed a high expression level in the S-G cells but showed lower expression than other *let-7* homologs in the NPC cells. Upon treatment with NGN, S-G cells showed a slight increase in *let-7g*, whereas NPC cells presented profoundly elevated *let-7g* levels (Figure 4A). Previous results show that protein kinase C (PKC) activity regulates *let-7g* expression by increasing the OCT-1 transcription factor [47]. Therefore, a specific PKC inhibitor chelerythrine (CHE), was used to validate *let-7g* up-regulation. The inhibition of PKC by CHE had no effects on *let-7g* and *OCT-1* expressions (Figure 4B,C), indicating that PKC does not act as an upstream regulator of OCT-1 modulating *let-7g* expression in the NPC and S-G cells.

To confirm whether *let-7g* upregulation leads to a reduction in KRAS and whether it affects the regulatory effect of B cell lymphoma 2 (BCL-2)/ B-cell lymphoma-extra large (BCL-x_L_) on mitochondrial function, *let-7g* mimic and *let-7g* inhibitor were utilized to validate the abovementioned questions. The overexpression of the negative (N) mimic control did not influence the signaling and metabolic parameters of NPC cells, whereas *let-7g* mimic expression downregulated KRAS expression and caused impacts similar to those of NGN on the decreased levels of p-Akt (Ser 473), GTP-Rac1, mitochondrial OCR, ECAR, glucose uptake, intracellular glucose, pyruvate, lactate, and ATP (Figure 4F–O and Supplementary Tables S3 and S4). In line with NGN treatment, *let-7g* mimic also resulted in the induction of cyclin B1, cyclin dependent kinase 1 (CDK1) (Thr 161) phosphorylation, BCL-2 (Thr 69/Ser 87) phosphorylation, BCL-x_L_ (Ser 62) phosphorylation, procaspase-3/-9/-12 cleavage, pro-apoptotic Bcl-2-associated x protein (BAX)/Bcl-2 antagonist killer 1 (BAK) oligomerization on the endoplasmic reticulum (ER)/mitochondria, cytosolic calcium (Ca^++^), and mitochondrial reactive oxygen species (ROS) (Figure 4E,F,I,J). The proteolytical process of Bid could not be triggered by NGN or *let-7g* mimic (Figure 4F). Compared with the (N) control inhibitor, *let-7g* inhibitor effectively inhibited endogenous *let-7g* expression and repressed the NGN-induced suppressive events; meanwhile, the activities of glucose uptake, cytosolic Ca^++^, mitochondrial ROS, glycolysis, mitochondrial OXPHOS, and ATP production recovered similar to those of vehicle-treated cells (Figure 4E–O and Supplementary Tables S4 and S5). Mitoquinone (MitoQ), a mitochondria-targeted antioxidant, completely inhibited NGN-induced mitochondrial ROS generation, whereas the non-antioxidant control decyltriphenylphosphonium bromide (DecylTPP) did not result in any suppression (Figure 4I). MitoQ partially inhibited the NGN-induced increase in cytosolic Ca^++^. Elevated Ca^++^ and ROS levels were ultimately be inhibited by dantrolene, an inhibitor of Ca^++^ channel proteins. The concentration of cytosolic Ca^++^ was higher than that of NGN-treated cells with the addition of ruthenium red, an inhibitor of mitochondrial Ca^++^ uptake (Figure 4J). However, the induction of mitochondrial ROS by NGN was significantly attenuated by ruthenium red (Figure 4I,J), indicating that NGN-induced ER-mitochondrial Ca^++^ flux contributed to mitochondrial oxidative stress. In contrast, S-G cells’ survival, OCR, and ECAR were unaffected by N mimic, *let-7g* mimic, or *let-7g* inhibitor overexpression, similar to NGN treatment (Supplementary Figure S1). These results indicate that impairment of the KRAS–PI3K–Rac1–Akt-modulated mitochondrial bioenergetics in NPC cells resulted from *let-7g*-suppressed KRAS expression.

### 3.4. Let-7g Upregulation-Induced Suppression of Lipid Raft-Associated GLUT-1 Is Involved in the Inhibition of Glycolytic and Mitochondrial OXPHOS Activity

**Figure 5:**
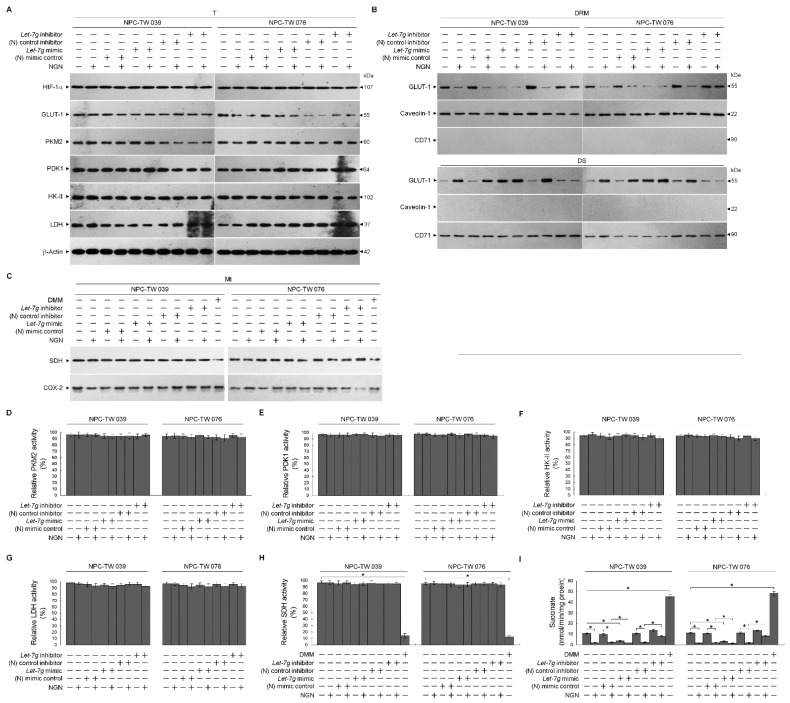
*Lethal-7g* (*let-7g*) upregulation attenuates glucose transporter-1 (GLUT-1) lipid raft membrane-targeting without affecting hypoxia-inducible factor 1α (HIF-1α)-mediated pyruvate kinase type M2 (PKM2), pyruvate dehydrogenase kinase 1 (PDK1), hexokinase II (HK-II), lactate dehydrogenase (LDH), and succinate dehydrogenase (SDH) activities. (**A**–**I**) At 12 h after transfection with the negative (N) mimic control, *let-7g* mimic, (N) mimic control inhibitor, or *let-7g* inhibitor, cells treated with vehicle (−) or naringenin (NGN) for 36 h. The levels of the indicated proteins in the total cell (T) lysates or detergent-resistant membrane (DRM) fractions were determined by Western blot analysis using specific antibodies. The FKM2, PDK1, HK-II, LDH, and SDH activities were analyzed using the Colorimetric-Based Pyruvate Kinase Activity Assay, ADP-GloTM Kinase Assay, Colorimetric Hexokinase Activity Assay, Lactate Dehydrogenase Assay, and Succinate Dehydrogenase Activity Colorimetric Assay, respectively. The Succinate Colorimetric Assay kit determined the succinate level. β-Actin was used as an internal control for sample loading. * *p* < 0.05.

Enhanced hypoxia-inducible factor 1α (HIF-1α)-mediated pyruvate kinase type M2 (PKM2), pyruvate dehydrogenase kinase 1 (PDK1), and glucose transporter-1 (GLUT-1) expression were found to regulate the energy metabolism of NPC cells [48]. NPC cells increase growth and invasive potential by inducting HIF-1α-mediated hexokinase II (HK-II) activity [49]. Succinate dehydrogenase (SDH) is one of the hubs linking oxidative phosphorylation and electron transport to generate ATP [50]. To this end, we investigated the expression of these proteins and an essential enzyme lactate dehydrogenase (LDH) of the anaerobic metabolic pathway [51]. The HIF-1α, GLUT1, PKM2, PDK1, HK-II, LDH, and SDH levels were unaffected by NGN, (N) mimic control, *let-7g* mimic, (N) control inhibitor, or *let-7g* inhibitor treatment: moreover, these enzyme activities were not altered by the above condition (Figure 5A,C–H). Dimethyl malonate (DMM), an inhibitor of SDH to prevent the oxidation of succinate to fumarate [52], has a specific inhibitory effect on the SDH activity and increases the succinate level (Figure 5H,I). The transfection of *let-7g* mimic resulted in an inhibitory effect on succinate that was similar to that of NGN. *Let-7g* inhibitor reversed the decrease in succinate caused by NGN (Figure 5I). The GLUT-1 level in the lipid rafts decreased by NGN or *let-7g* mimic treatment (Figure 5B). *Let-7g* inhibitor addition abolished the decrease in GLUT-1 in the lipid rafts of cells stimulated with NGN (Figure 5B). These results indicate that the attenuation of GLUT-1 lipid raft membrane-targeting by *let-7g* upregulation contributed to impeding the metabolic functions of glycolysis and mitochondrial OXPHOS.

### 3.5. NGN Inhibited the MCPIP1-Mediated Degradation of let-7g

**Figure 6:**
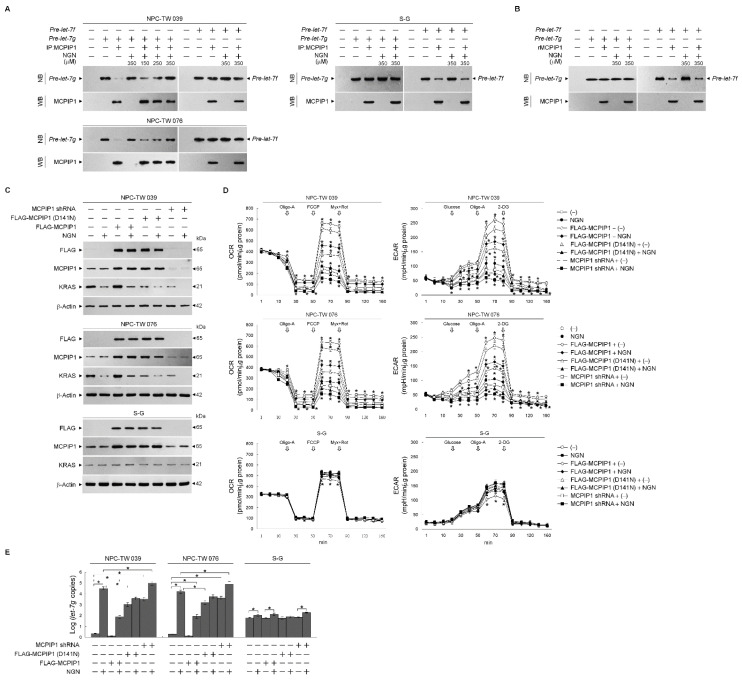
Suppression of monocyte chemoattractant protein-induced protein-1 (MCPIP1)-mediated degradation of *lethal-7g* (*let-7g*) by naringenin (NGN) involved the attenuation of glycolysis and mitochondrial mitochondrial oxidative phosphorylation (OXPHOS). (**A**,**B**) Immunoprecipitated MCPIP1 (IP:MCPIP1) from the nasopharyngeal carcinoma (NPC) or Smulow–Glickman (S-G) cells and recombinant purified MCPIP1 (rMCPIP1) used in the experiments. The inhibition of the *let-7g* degradation of MCPIP1-mediated by NGN, performed by in vitro RNA cleavage assay and analyzed by Northern blotting (NB). The levels of the IP:MCPIP1 or rMCPIP1 in reactions were determined by Western blot (WB) analysis using specific antibodies. (**C**–**E**) At 12 h after transfection with FLAG-MCPIP1, FLAG-MCPIP1 (D141N), or MCPIP1 short hairpin RNA (shRNA), cells were treated with vehicle (−) or NGN in the presence of 1 μM oligomycin-A (Oligo-A), carbonylcyanide-p-trifluoromethoxyphenylhydrazone (FCCP), and rotenone (Rot) plus myxothiazol (Myx) or the sequential addition of glucose, Oligo-A, and 2-deoxy-D-glucose (2-DG) at the indicated time points. The oxygen consumption rate (OCR) and extracellular acidification rate (ECAR) were measured using a Seahorse Bioscience XF24 Analyzer. Western blot analysis using specific antibodies determined the levels of the indicated proteins in the total cell lysates. Quantitative real-time polymerase chain reaction (qRT-PCR) determined the relative expression level of *let-7g.* The *let-7g* value was normalized to the U6 level. The Y-axis shows the denary logarithm of the normalized *let-7g* copy number. The values are presented as three independent experiments’ mean ± standard error. * *p* < 0.05: significantly different from (−)-treated empty vector-transfected, NGN-treated empty vector-transfected, (−)-treated FLAG-MCPIP1-transfected, or (−)-treated MCPIP1 shRNA-transfected cells.

The ribonucleolytic cleavage of *pre-let-7g* by monocyte chemoattractant protein-induced protein-1 (MCPIP1) implicates the regulation of *let-7g* biogenesis [37]. Therefore, to address whether targeting of NGN to MCPIP1 reflects the elevation of *let-7g*, MCPIP1 proteins were immunoprecipitated from the lysates of NPC-TW039, NPC-TW076, or S-G cells with an antibody to MCPIP1. Immunoprecipitated MCPIP1 (IP:MCPIP1) from the NPC cells degrades *pre-let-7g* but not *pre-let-7f*. The preparation of IP:MCPIP1 from the S-G cells or recombinant purified MCPIP1 (rMCPIP1) did not degrade *pre-let-7g*; it caused the cleavage of *pre-let-7f* (Figure 6A,B). NGN revealed an inhibitory effect on the degradation of *pre-let-7g* by NPC-derived IP:MCPIP1 (Figure 6A); however, it did not abolish the ribonuclease cleavage of S-G-derived IP:MCPIP1 or rMCPIP1 to *pre-let-7f* (Figure 6A,B). The overexpression of FLAG-tagged MCPIP1 (FLAG-MCPIP1) in the NPC cells significantly reduced *let-7g*, increasing KRAS, OCR, and ECAR. Moreover, FLAG-MCPIP1 could overcome the suppression of the OCR and ECAR by NGN. Elevated *let-7g* by FLAG-MCPIP1 (D141N) (lacking the RNase activity) or MCPIP1 shRNA caused the elevation of *let-7g* and a decrease in KRAS, OCR, and ECAR, similar to the effects of NGN treatment. In the S-G cells, FLAG-MCPIP1 overexpression did not affect the level of *let-7g* but decreased the OCR and ECAR. NGN could partially reverse the suppression effects of FLAG-MCPIP1 expression on the OCR and ECAR. FLAG-MCPIP1 (D141N) or MCPIP1 shRNA overexpression did not affect the level of *let-7g* (Figure 6C–E). These results suggest that decreased OCR and ECAR levels were associated with the suppression of the MCPIP1-mediated degradation of *let-7g* by NGN in the NPC cells.

## 4. Discussion 

By dysregulating proliferative signaling pathways, cancer cells with alternative metabolism can supply the energy required for continuous growth, rapid proliferation, and the malignant phenotype. The essential organelle for maintaining the bioenergetics of cancer cells is the mitochondria [7,53]. Targeting the signal regulators required for the mitochondria’s metabolic bioenergetics is a promising strategy for cancer therapy [7]. Although the aberrant activation of PI3K–Rac1–Akt signaling acts as a crucial coordinator of glycolytic and ER–mitochondrial metabolic functions to conduct the metabolic reprogramming of NPC cells by regulating the lipid raft-associated GLUT-1-mediated glucose uptake [30,54], the precise regulator mechanism of PI3K activation remains unclear. Our findings indicate that KRAS colocalizes and complexes with p110α, p85α, and GTP-Rac1 in the lipid rafts of NPC cells. The expression of KRAS shRNA in NPC cells resulted in a decrease in the levels of various critical cellular components and activities, including lipid raft-associated p85α–p110α–GTP-Rac1 complexes, Akt phosphorylation, glucose metabolism, and mitochondrial function. These findings suggest that KRAS substantially impacts NPC cell’s metabolic biology and function. The pro-survival metabolic signal emanates from PI3K–Rac1–Akt conducted by KRAS. The study of the *KRAS*-specific mRNA silencing activity of *let-7g* in human lung and hepatocellular carcinoma cells [14,15], together with *let-7g* as one of the dysregulated miRNAs in NPC tissues and close correlation with survival time of NPC patients [12], in conjunction with the expression of HA-KRAS in NPC cells promoting the formation of lipid raft-associated p85α–p110α–GTP-Rac1 complexes, glucose uptake, and metabolic activity and ultimately influencing the cellular physiology of nasopharyngeal carcinoma cells, suggests that the dysregulation of *let-7g* might be associated with the progression of NPC by allowing the upregulation of *KRAS*. We observed that endogenous *let-7g* had a high expression level in S-G cells and lower expression in NPC cells than other *let-7* homologs, indicating that NPC cells may be a specific modulator in regulating *let-7g* expression. The enforced expression of *let-7g* mimic had significant inhibitory effects on the PI3K−Rac1−Akt−BCL-2/BCL-x_L_-modulated metabolic and apoptotic signaling pathways in NPC cells by downregulating KRAS. *Let-7g* inhibitor effectively inhibited endogenous *let-7g* expression, increasing the KRAS level in NPC cells, and this led to the elevation of p-Akt (Ser 473), intracellular glucose, and OCR and ECAR metabolic parameters and the decline of cytosolic Ca^++^ and mitochondrial ROS levels. However, these effects were not observed in S-G cells, which appeared to be more resistant to the influence of *let-7g* mimic and inhibitor expression. The induction of p-CDK1 (Thr 161)–cyclin B1 activity in NPC cells relates to downregulation of the PI3K–Rac1–Akt pathway [30]. An elevated level of cyclin B1 accounts for the formation of CDK1–cyclin B1 complexes [55]. Thr 161 phosphorylation is critical for maintaining CDK1 kinase activity and initiates this by binding the cyclin B1 to CDK1 [55,56,57]. Unscheduled CDK1–cyclin B1 activity can trigger BCL-2 (Thr 69 and Ser 87)/BCL-x_L_ (Ser 62) phosphorylation and mitochondria-ER-targeted BAX/BAK oligomerization, which causes abnormal oxidative stress and impair ER–mitochondrial bioenergetics [30]. The disability of BCL-2/BCL-x_L_ to suppress the BAX/BAK pro-apoptotic function in the NPC cells occurs due to the initiation of p-CDK1 (Thr 161)–cyclin B1-induced BCL-2 (Thr 69 and Ser 87)/BCL-x_L_ (Ser 62) phosphorylation [30]. Consistent with the previously demonstrated findings, increased apoptosis is related to the down-regulation of lipid raft-associated GLUT-1 caused by PI3K–Rac1–Akt inactivation [30]. These observations indicate that *let-7g* emerges as a significant target in NPC tumors due to the regulation of KRAS expression and its downstream PI3K−Rac1−Akt−BCL-2/BCL-x_L_-mediated bioenergetic and apoptotic signaling pathways. *Let-7g* dysregulation in NPC tissues and the functional impact on NPC cells underscore its potential relevance in the clinical context of NPC, and it may have implications for the progression of NPC tumors in the in vivo environment. Further research is needed to fully elucidate the in vivo effects of *let-7g* dysregulation in NPC.

The expression of MCPIP1 shRNA or FLAG-MCPIP1 (D141N) in the NPC cells, resulting in the elevation of *let-7g,* caused the loss of KRAS-modulating glycolysis and mitochondrial respiration functions; however, FLAG-MCPIP1 overexpression led to the decline of *let-7g*, facilitating KRAS-mediated glycolysis and mitochondrial respiration activities. In S-G cells, the overexpression of FLAG-MCPIP1 did not significantly affect the level of *let-7g*. However, it did decrease the OCR and ECAR. FLAG-MCPIP1 (D141N) or MCPIP1 shRNA overexpression did not significantly impact the level of *let-7g* in S-G cells. Immunoprecipitated MCPIP1 (IP:MCPIP1) from NPC cells degraded *pre-let-7g* but not *pre-let-7f*. In contrast to NPC cells, the preparation of IP:MCPIP1 from S-G cells or recombinant purified MCPIP1 (rMCPIP1) did not degrade *pre-let-7g*; instead, it caused the cleavage of *pre-let-7f*. The use of *Caenorhabditis elegans* as a model to study the functionally essential structural features of MCPIP1-like ribonuclease RDE-8 indicates that argonaute RDE-1 can recruit RDE-8 to guide RDE-8 and it-associated proteins to small interfering RNA targets [58]. These observations suggest that the association of argonaute protein or an undefined cofactor may achieve sequence-specific targeting of *pre-let-7g* and help to control MCPIP1-mediated *pre-let-7g* degradation in NPC cells. Thus, MCPIP1-modulated *let-7g* influences the metabolic activities crucial for NPC cell growth. However, MCPIP1 does not significantly impact *let-7g* levels in normal human gingival epithelial cells. The mechanism of *let-7g* regulation by MCPIP1 is cell specific, suggesting a potential therapeutic target for NPC without affecting normal cells. Further research is needed to fully understand the precise molecular mechanisms and potential clinical applications.

**Figure 7:**
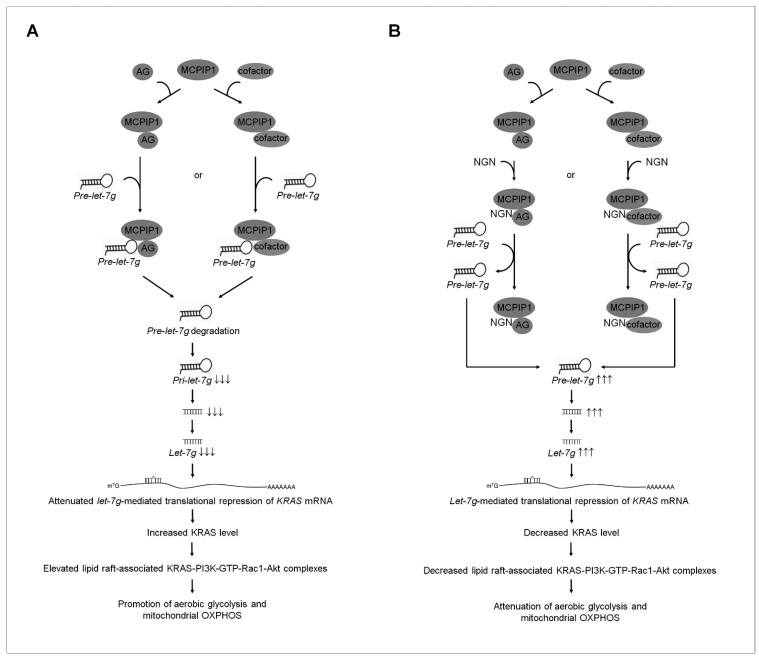
A molecular model for the naringenin (NGN)-induced impairment of the mitochondria-mediated bioenergetics in nasopharyngeal carcinoma (NPC) cells. (**A**) The selective interaction of argonaute (AG) protein or an undefined factor with monocyte chemoattractant protein-induced protein-1 (MCPIP1) may accomplish the sequence-specific targeting of *pre-lethal-7g* (*pre-let-7g*) and promote MCPIP1-mediated *pre-let-7g* degradation to decrease the biogenesis of *let-7g*, resulting in the attenuation of the *let-7g*-mediated translational repression of kirsten rat sarcoma viral oncogene homolog (*KRAS*) mRNA. The resultant elevated KRAS increases the formation of clustered KRAS- phosphatidylinositol 3-kinase (PI3K)-active GTP-binding Ras-related C3 botulinum toxin substrate (GTP-Rac1)–protein kinase B (Akt) signaling molecules in the lipid raft membranes, constituting a central element in the initiation of the coordination of glycolysis with mitochondrial oxidative phosphorylation (OXPHOS) for ATP generation. (**B**) Under the condition of the cellular uptake of NGN, NGN may bind to the AG protein or an undefined cofactor to block the degradation of *pre-let-7g* by MCPIP1, increasing the level of *let-7g*, thus inducing the *let-7g*-mediated translational repression of *KRAS* mRNA and thereby dismissing the interaction between KRAS and p110α in the lipid raft membrane. The absence of KRAS in the p85α–p110α complexes causes the destabilization of p85α–p110α complexes in the lipid raft membrane. The resultant loss of the KRAS–p85α–p110α complexes in the lipid raft membrane leads to blocking PI3K-GTP-Rac1-mediated Akt activation. Attenuated Akt impaired the aerobic glycolysis and mitochondria-regulated bioenergetic functions in NPC cells.

Ectopically expressed Epstein–Barr virus (EBV) latent membrane protein 1 (LMP1) in an EBV-negative NPC cell line (NPC-TW01) enhanced glycolysis rather than mitochondrial OXPHOS [48]. NPC cells’ selectivity towards aerobic glycolysis depends on LMP1-mediated HIF-1α activation and the increased expression of glycolytic enzymes (PKM2, PDK1, HK-II, and LDH) [48]. Further studies showed that the ectopic expression of LMP1 in the EBV-negative NPC cell line reduces oxygen consumption and mitochondrial membrane potential [48]. HIF-1α-mediated PDK1 activation results in the preference of cancer cells towards aerobic glycolysis. Furthermore, HIF-1 *trans*-activates PDK-1 expression, and PDK1 inactivates pyruvate dehydrogenase, resulting in the inhibition of pyruvate entry into the TCA cycle, which in turn suppresses metabolism [59,60]. HIF-1α expression and the expression levels and enzyme activities of PKM2, PDK1, HK-II, LDH, and SDH were not affected by the *let-7g* mimic-mediated attenuation of KRAS expression; however, *let-7g* mimic resulted in impaired functions of glycolysis and mitochondrial OXPHOS. These observations explain the results of the present study because EBV-negative NPC-TW 039 and NPC-TW 076 cells exhibit coordinated glycolysis and mitochondrial OXPHOS for ATP generation. KRAS−PI3K−Rac1−Akt−BCL-2/BCL-x_L_-mediated signaling conducts glycolysis and mitochondrial OXPHOS activities in EBV-negative cells. Growing evidence from the studies of in vitro cell line modes and in vivo tissue analysis indicated that flavonoids, a group of natural substances with variable phenolic structures, can modulate miRNA expression, suppressing the growth of human cancer cells [61]. The findings indicate that NGN, a flavonoid compound, induces increased *let-7g* expression, thus attenuating the oncogenic KRAS-modulated metabolic activity. The quantitative analysis of the qRT-PCR showed that *let-7g* shows a lower expression level than its family of *let-7* gene homologs. Further studies revealed selectively increased *let-7g* expression in NGN-treated NPC cells. A recent study using human hepatoma HepaRG cells found that NGN suppressed the activity of oncogenic metabolism through the regulation of miRNA-targeted mRNA expression inhibiting PI3K–Akt signaling [62]. The reported evidence indicates that the expression of has-miR-1306-5p, has-miR-627-3p, has-miR-194-3p, has-miR-676-3p, has-miR-6837-5p, has-miR-429, has-miR-100-3p, has-miR-194-5p, has-miR-519a-3p, has-miR-7-5p, and has-miR-200a-5p was involved in the attenuation of the PI3K–Akt signaling pathway of HepaRG cells by NGN [62]. The involvement of NGN-induced miRNAs in the negative regulation of PI3K–Akt-mediated anti-oxidative stress and ameliorative metabolism was postulated to be associated with the down-regulation of platelet-derived growth factor receptor β, phosphoenolpyruvate carboxykinase 1, cAMP-responsive element-binding protein 3 like 1, and nuclear factor kappa B mRNA expression targeted by has-miR-1306-5p or has-miR-627-3p [62]. However, this observation is inconsistent with our model of the upregulation of *let-7g* by NGN-attenuated KRAS–PI3K–Rac1–Akt axis metabolic functions, probably because the study cited used a hepatoma cell that conferred the cell-type specificity of miRNA expression to post-translational regulation of the expression of PI3K–Akt-regulated mRNAs involved in metabolism. Studies from chronic myelogenous leukemia [63], breast cancer [64], colon cancer [65], and glioblastoma cells [66] showed the pharmacological activities of NGN by targeting multiple signal pathways, indicating that NGN could differentially modulate the expression of cellular genes that are involved in cell growth, apoptosis, and cell invasion in different types of cancer cells. Although NGN induced *let-7g* expression and decreased KRAS, apoptosis and bioenergetic dysfunction were not observed in the S-G cells. Further evidence has indicated that NGN did not affect p85α–p110α complex formation but attenuated p-Akt (Ser 473) levels in the lipid rafts of S-G cells. Consistent with the previous findings in normal human cells [67,68,69], p85α expressed a relatively high protein level compared to p110α in the S-G cells. p85α, predominantly monomeric form at high concentration, is thought to be the major effector of response to growth stimulation and acts as a negative regulator of the PI3K-Akt signal pathway to control the proliferation and survival of normal human cells [67,68,69,70,71,72]. Phosphorylated Akt (Ser 473) and activated GTP-Rac1 were present in the S-G cells, whereas GTP-Rac1 and KRAS did not form a complex with p85α–p110α in the lipid rafts. Functional analyses demonstrated that gain-of-function mutations in p110α confer the ability to interact with KRAS, forming a complex responsible for the enhanced oncogenic activity of cancer cells [18,73]. Targeting *let-7g*-mediated KRAS suppression explains why the loss of the inhibitory effect of NGN on the PI3K-Akt-regulated bioenergetics in normal human cells may be due to the lack of oncogenic mutation in the catalytic subunit p110α of PI3K, which decreases the interaction with KRAS. Studies of in vitro RNA cleavage assay using NPC-derived IP:MCPIP1, S-G-derived IP:MCPIP1, or rMCPIP1 have suggested argonaute protein or an undefined cofactor is involved in the RNA specificity to MCPIP1-mediated *pre-let-7g* cleavage. NGN could effectively block the degradation of *pre-let-7g* by NPC-derived IP:MCPIP1. No inhibitory effect of NGN on the S-G-derived IP:MCPIP1 or rMCPIP1-caused *pre-let-7f* degradation was observed. NPC cells overexpressing FLAG-MCPIP1 overcome NGN-induced alterations in KRAS, OCR, and ECAR levels. Using a pharmacological inhibition strategy in which CHE inhibited PKC activity, we have demonstrated here that the regulation of *let-7g* is not required for initiation by PKC in the NPC and S-G cells. NGN exerted the effect of elevated *let-7g* expression on the ER–mitochondrial impairment of bioenergetics towards binding to the argonaute protein or an undefined cofactor to interfere with MCPIP1-modulated *let-7g* biogenesis. Based on the above summarized and previous findings [30,33], with the evidence from the present study, we propose a working model whereby NGN can suppress the function of mitochondrial bioenergetics in NPC cells (Figure 7). NGN has a specific inhibitory effect on the metabolic activities of NPC cells, particularly in suppressing oncogenic KRAS−PI3K−Rac1−Akt−BCL-2/BCL-x_L_ signaling pathways by attenuating MCPIP1-mediated *pre-let-7g* degradation. It does not have the same impact on normal cells. However, it is essential to note that the specific effects of NGN on different types of cancer cells and their microenvironments may vary, and further research is needed to understand its therapeutic potential and potential side effects fully. Additionally, the possible broader impacts of NGN on other cell types or tumor microenvironments would require more extensive investigation. Both male (NPC-TW 039) and female (NPC-TW 076) human NPC cell lines exhibited similar responses to NGN in terms of bioenergetic inhibition and the induction of apoptosis. These effects were consistent across the sexes, suggesting that sex differences did not influence the sensitivity of NPC cells to NGN in this study. Notably, our findings have implications for resolving the molecular mechanism of NGN repression of NPC cell bioenergetics. Understanding the function of *let-7g* as a KRAS-repressing molecule makes it an essential target for developing therapeutic strategies for human cancers in which KRAS is overexpressed.

## 5. Conclusions

The present study’s results represent a significant advancement in understanding the modulation of ER−mitochondrial bioenergetics by the MCPIP1-modulated *let-7g*–KRAS–PI3K–Rac1–Akt signaling axis and provide a mechanistic explanation for the role of KRAS as a crucial regulator of metabolic reprogramming towards the coordination of aerobic glycolysis and mitochondrial OXPHOS in the energy generation of NPC cells. The inhibition of MCPIP1-mediated *pre-let-7g* cleavage by NGN promotes the suppression of KRAS expression by *let-7g* upregulation, decreasing the formation of the lipid raft-associated KRAS–PI3K–GTP-Rac1 complex, thereby attenuating the PI3K–Rac1–Akt-mediated signaling. The inactivation of Akt leads to impaired glucose metabolism regulation by GLUT-1 and compromised mitochondrial bioenergetics governed by BCL-x_L_/BCL-2.

## Data Availability

All results generated or analyzed during present study are included in this published article. Data and materials will be made available upon request via email to the corresponding author (dr.chen3693@gmail.com).

## Supplementary Materials

The following supporting information can be downloaded at: https://www.mdpi.com/article/10.3390/cells12182313/s1, Figure S1: The effects of *let-7g* expression on the survival, OCR and ECAR of S-G cells; Table S1: Details of sources and concentrations of antibodies used for this study; Table S2.1: The effects of KRAS expression on the mitochondrial OCR of NPC-TW 039 cells; Table S2.2: The effect of KRAS expression on the mitochondrial OCR of NPC-TW 076 cells; Table S3.1: The effects of KRAS expression on the ECAR of NPC-TW 039 cells; Table S3.2: The effects of KRAS expression on the ECAR of NPC-TW 076 cells; Table S4.1: The effects of *Let-7g* expression on the OCR of NPC-TW 039 cells; Table S4.2: The effects of *Let-7g* expression on the OCR of NPC-TW 076 cells; Table S5.1: The effects of *Let-7g* expression on the ECAR of NPC-TW 039 cells; Table S5.2: The effects of *Let-7g* expression on the ECAR of NPC-TW 076 cells.

## Abbreviations

Adenosine triphosphate	ATP
B cell lymphoma 2	BCL-2
Bcl-2-antagonist of cell death	BAD
Bcl-2 antagonist killer 1	BAK
Bcl-2-associated x protein	BAX
BCL-2/B-cell lymphoma-extra large	BCL-x_L_
Cyclin dependent kinase 1	CDK1
Detergent-resistant membranes	DRM
Detergent-soluble	DS
Endoplasmic reticulum	ER
Epstein–Barr virus	EBV
Extracellular acidification rate	ECAR
Glucose transporter-1	GLUT-1
Hexokinase II	HK-II
Hypoxia-inducible factor 1α	HIF-α
Latent membrane protein 1	LAMP1
Lethal-7	let-7
MicroRNAs	miRNAs
Mitochondrial DNA	mtDNA
Mitochondrial oxidative phosphorylation	OXPHOS
Monocyte chemoattractant protein-induced protein-1	MCPIP1
Nasopharyngeal Carcinoma	NPC
Oxygen consumption rate	OCR
Phosphatase and tensin homolog deleted from chromosome 10	PTEN
Phosphatidylinositol 3-kinase	PI3K
Phosphatidylinositol-4,5-bisphosphate	PIP_2_
Phosphatidylinositol-3,4,5-trisphosphate	PIP_3_
Poly (ADP-ribose) polymerase	PARP
Protein kinase B	Akt
Protein kinase C	PKC
Pyruvate dehydrogenase kinase 1	PDK1
Pyruvate kinase type M2—	PKM2
Ras-related C3 botulinum toxin substrate 1	Rac1
Short hairpin RNA	shRNA
*Succinate dehydrogenase*	*SDH*
