# Supplementary figures and images for: Let-7g Upregulation Attenuated the KRAS–PI3K–Rac1–Akt Axis-Mediated Bioenergetic Functions
Source: Cells. 2023 Sep 19;12(18):2313. doi: 10.3390/cells12182313 (PMC10527334; doi:10.3390/cells12182313)

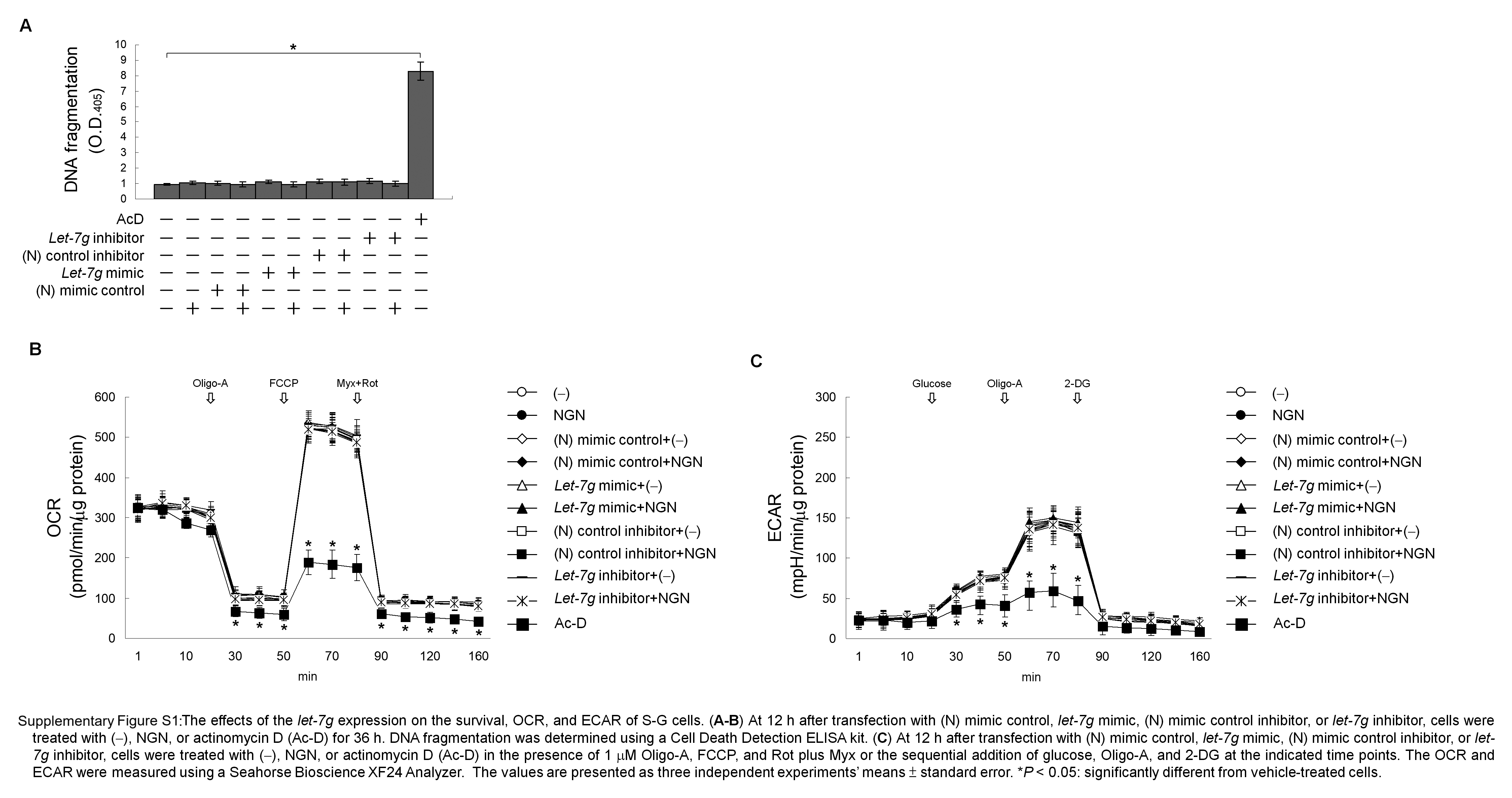

Supplement: Supplementary file 1 [file cells-12-02313-s001.zip › Figure S1.tif]

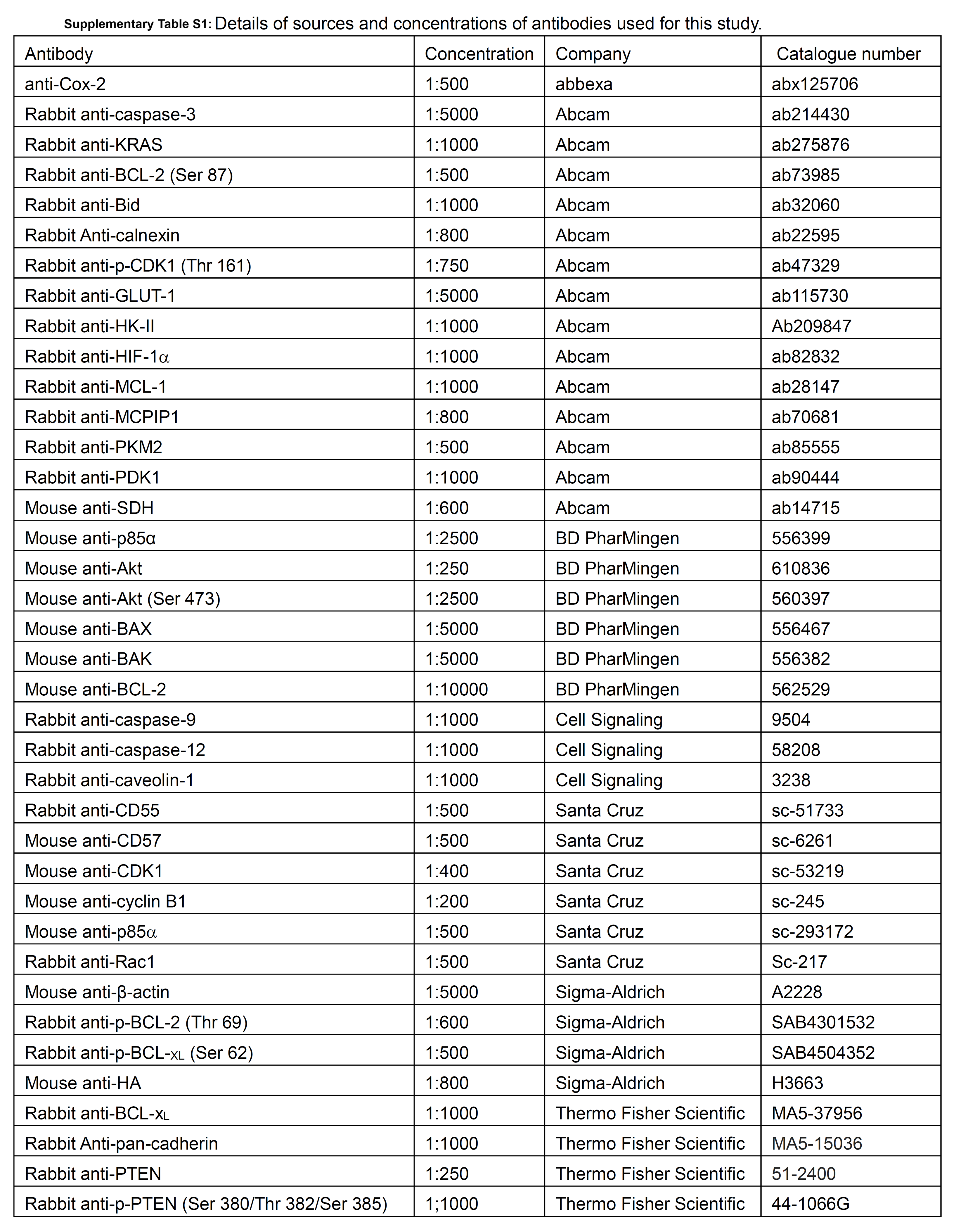

Supplement: Supplementary file 1 [file cells-12-02313-s001.zip › Table S1.tif]

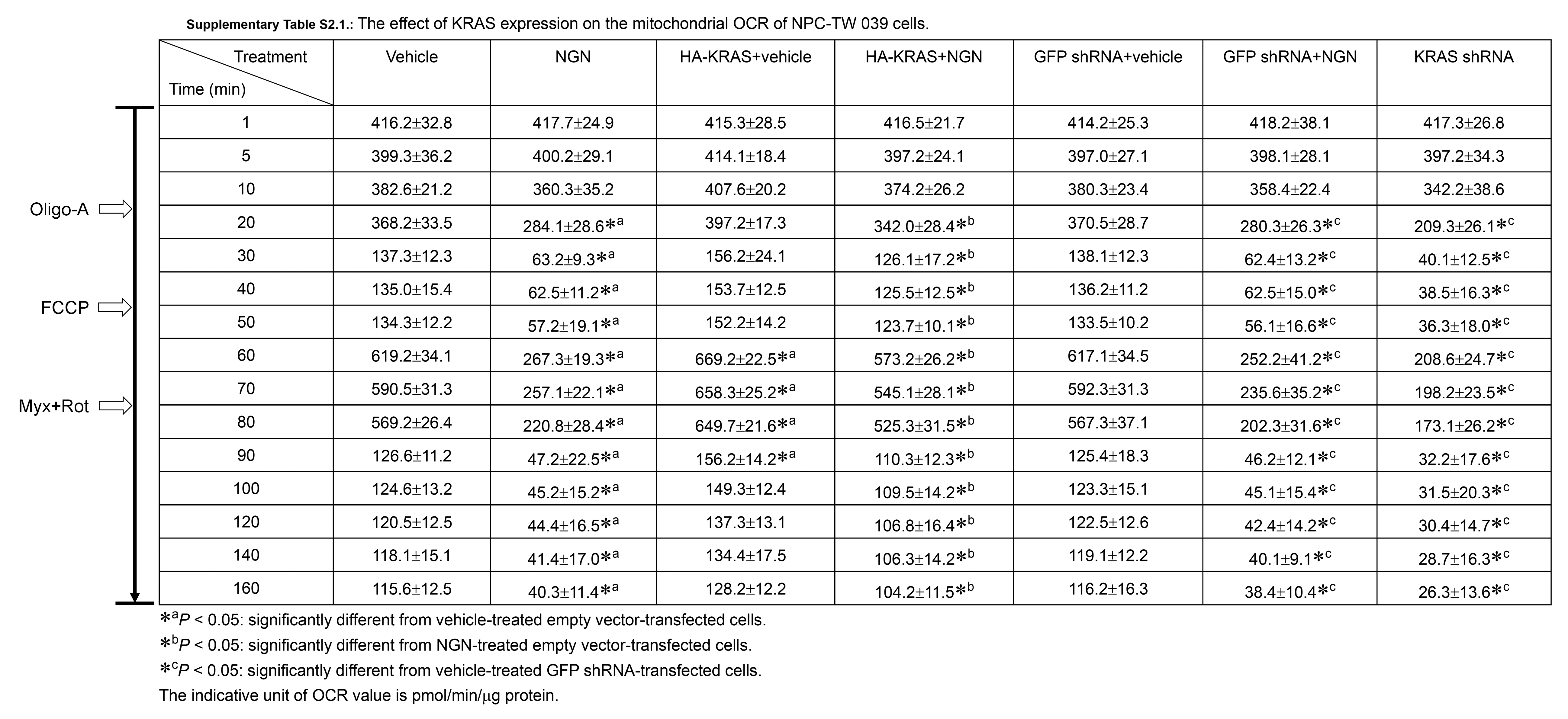

Supplement: Supplementary file 1 [file cells-12-02313-s001.zip › Table S2.1..tif]

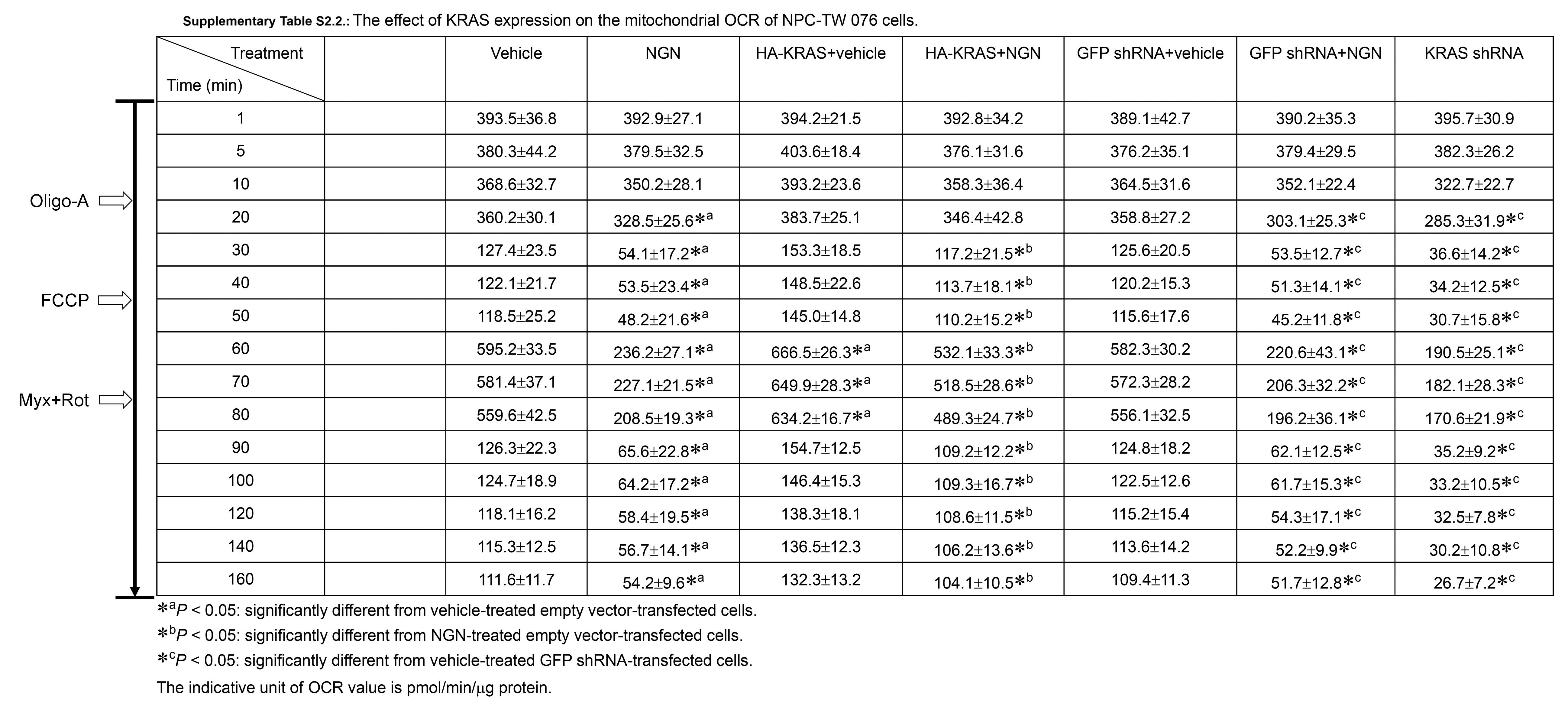

Supplement: Supplementary file 1 [file cells-12-02313-s001.zip › Table S2.2..tif]

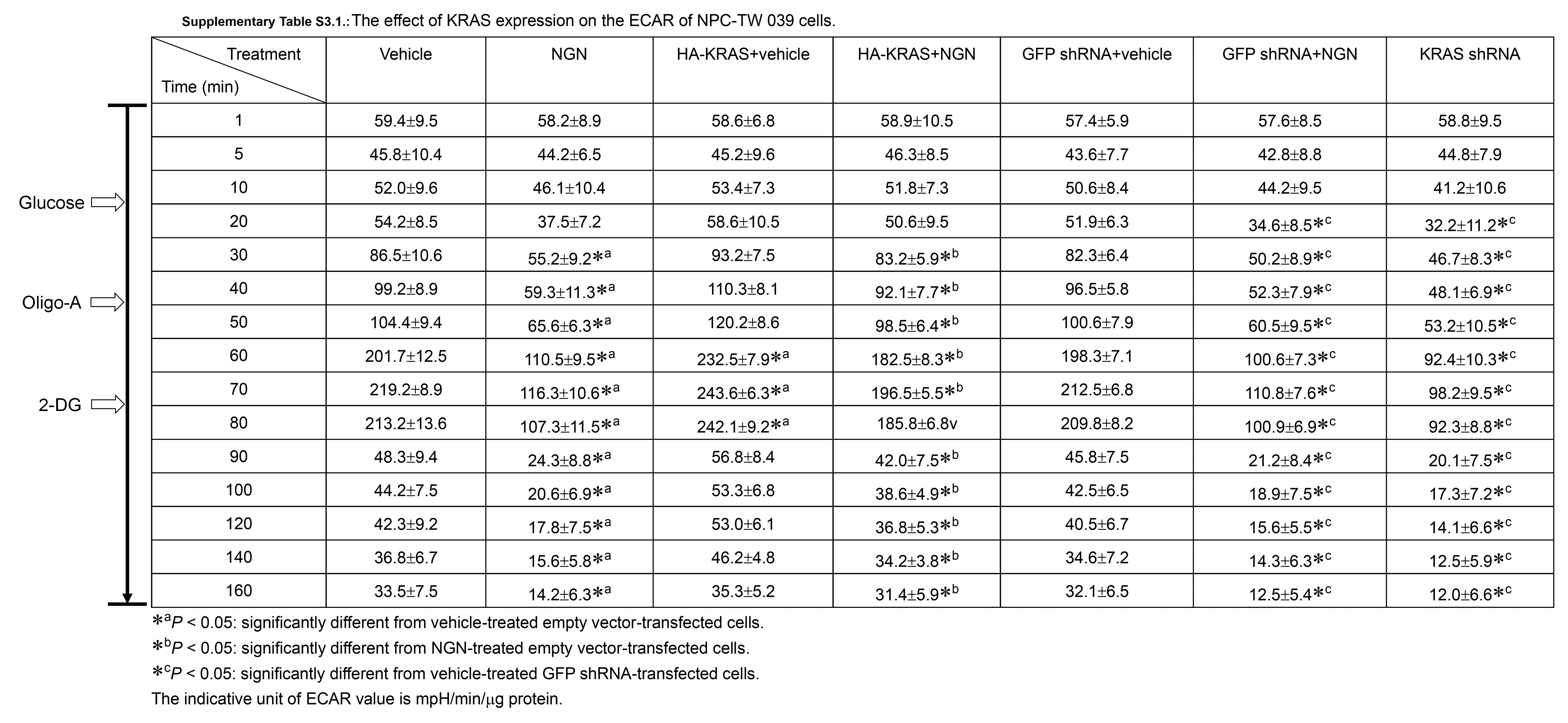

Supplement: Supplementary file 1 [file cells-12-02313-s001.zip › Table S3.1..tif]

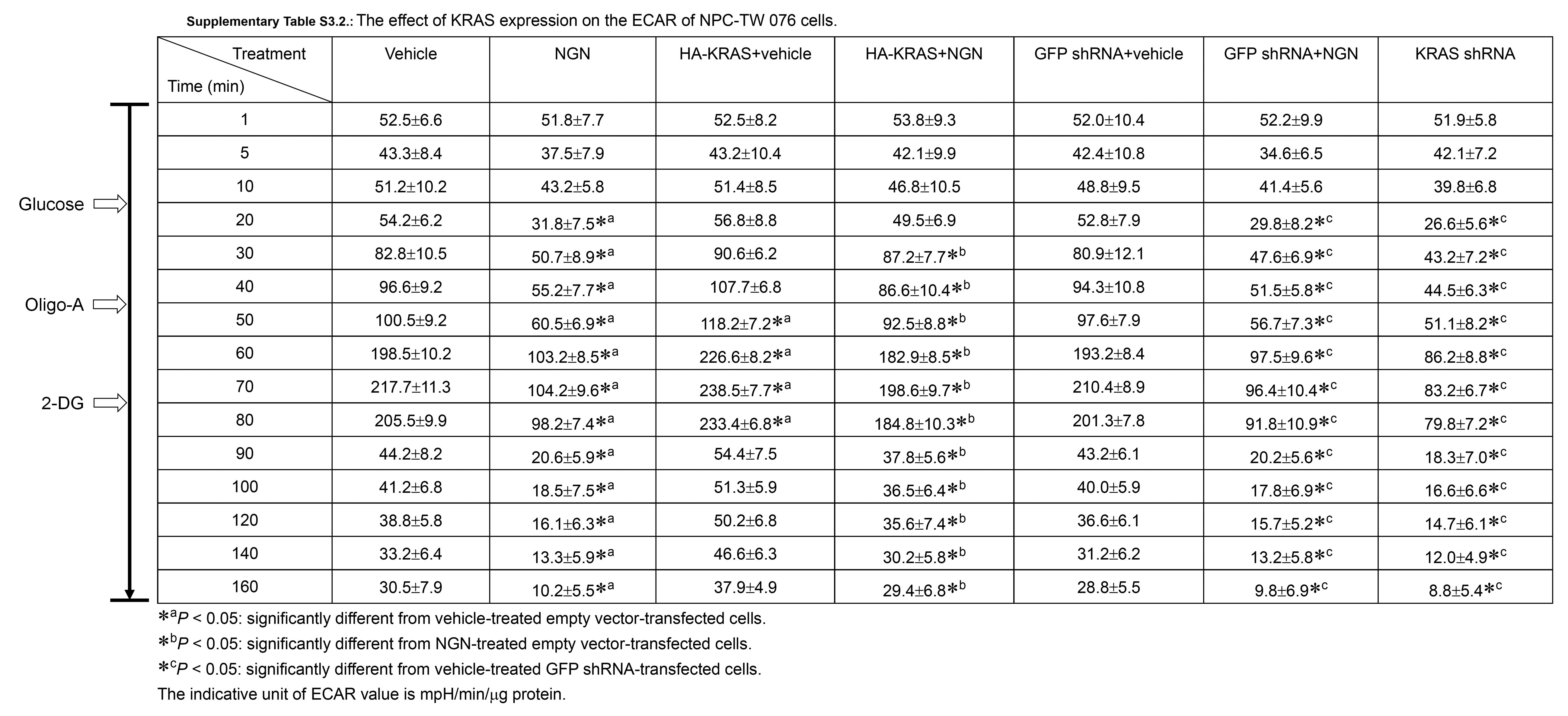

Supplement: Supplementary file 1 [file cells-12-02313-s001.zip › Table S3.2..tif]

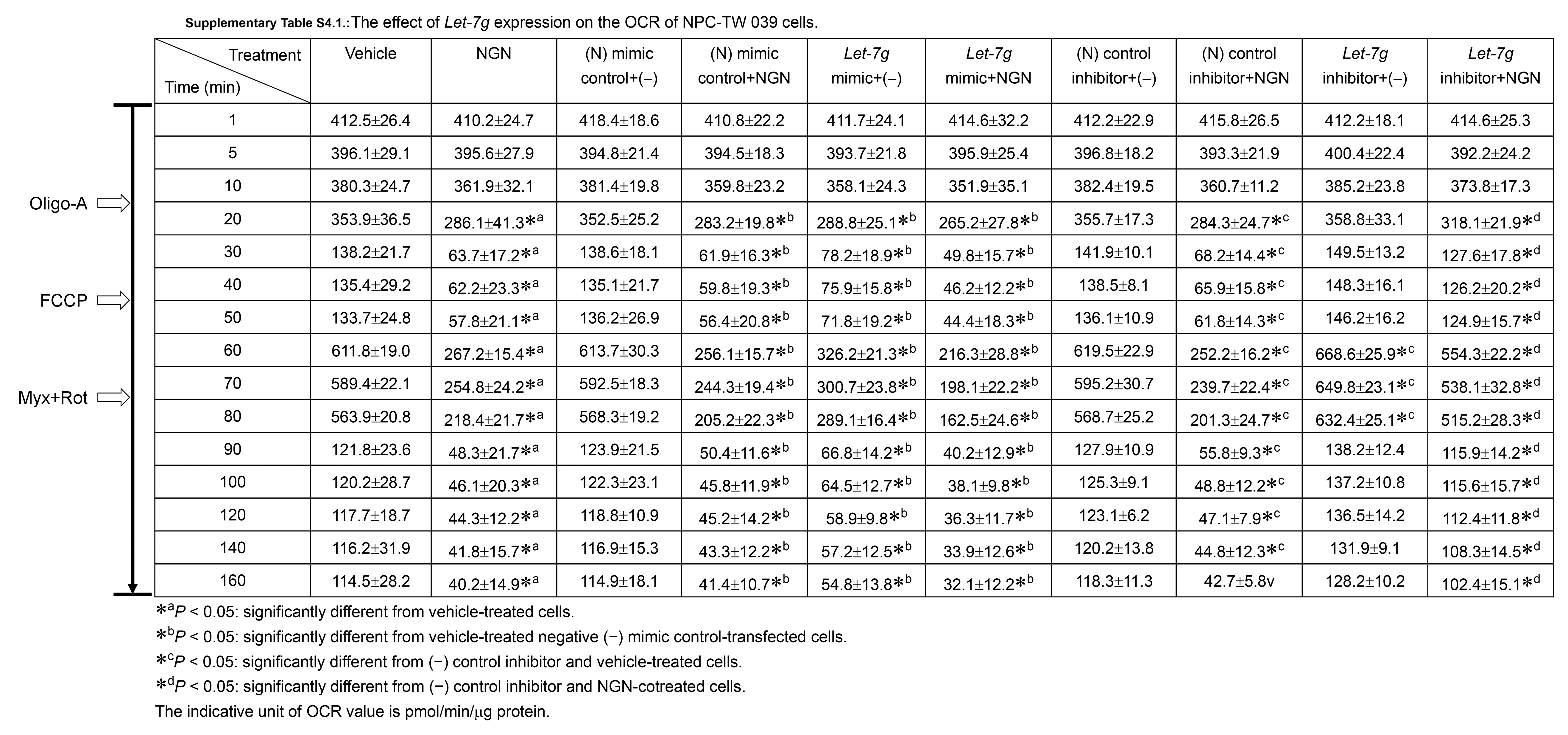

Supplement: Supplementary file 1 [file cells-12-02313-s001.zip › Table S4.1..tif]

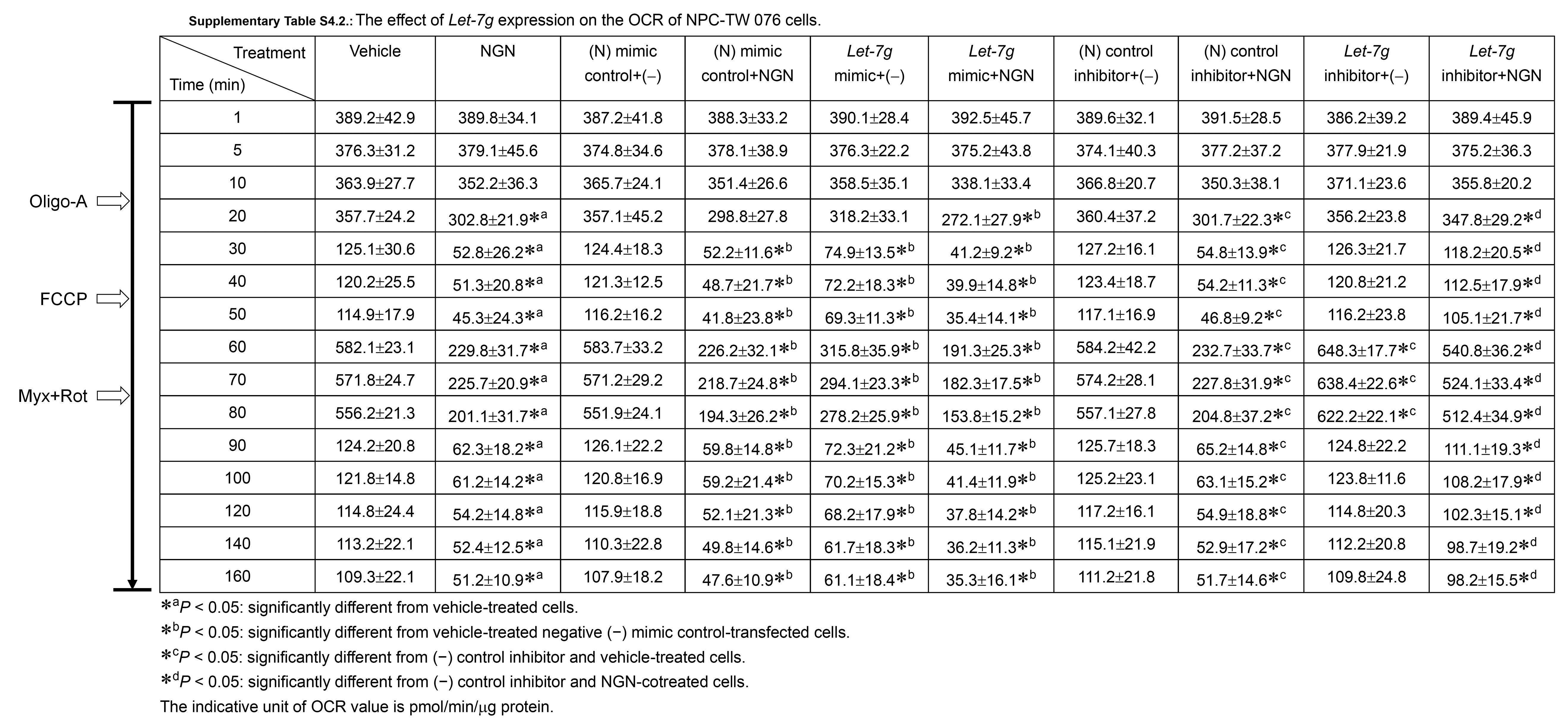

Supplement: Supplementary file 1 [file cells-12-02313-s001.zip › Table S4.2..tif]

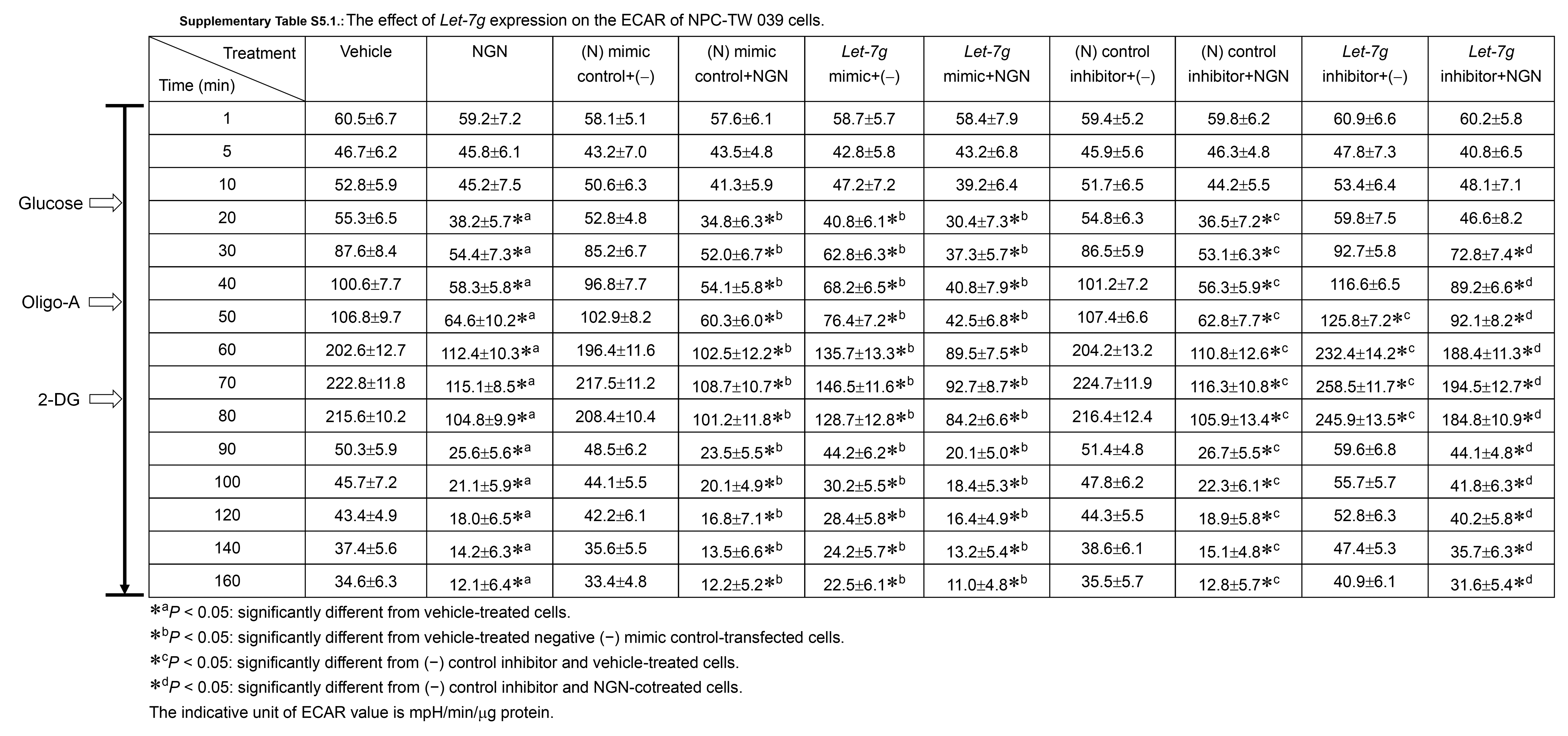

Supplement: Supplementary file 1 [file cells-12-02313-s001.zip › Table S5.1..tif]

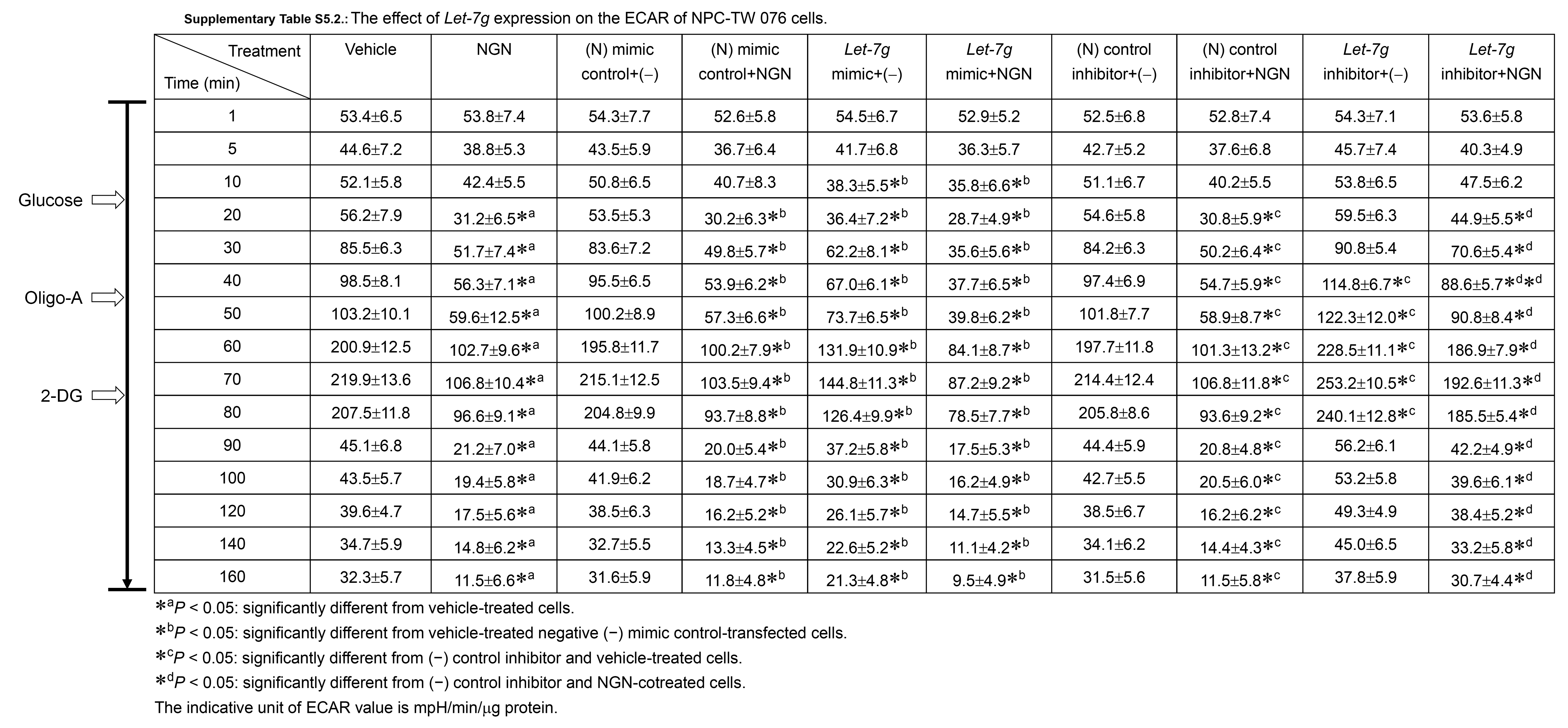

Supplement: Supplementary file 1 [file cells-12-02313-s001.zip › Table S5.2..tif]
